# Mitochondrial function controls intestinal epithelial stemness and proliferation

**DOI:** 10.1038/ncomms13171

**Published:** 2016-10-27

**Authors:** Emanuel Berger, Eva Rath, Detian Yuan, Nadine Waldschmitt, Sevana Khaloian, Michael Allgäuer, Ori Staszewski, Elena M. Lobner, Theresa Schöttl, Pieter Giesbertz, Olivia I. Coleman, Marco Prinz, Achim Weber, Markus Gerhard, Martin Klingenspor, Klaus-Peter Janssen, Mathias Heikenwalder, Dirk Haller

**Affiliations:** 1 Technische Universität München, Chair of Nutrition and Immunology, 85350 Freising-Weihenstephan, Germany; 2 Helmholtz-Zentrum München, Institute of Virology, 85764 Neuherberg, Germany; 3 Technische Universität München, Institute of Medical Microbiology, Immunology and Hygiene, 81675 Munich, Germany; 4 University of Freiburg, Institute of Neuropathology, Freiburg 79106, Germany; 5 Technische Universität München, Chair of Molecular Nutritional Medicine, Else Kröner-Fresenius Center, 85350 Freising-Weihenstephan, Germany; 6 Technische Universität München, Department of Nutritional Physiology, 85350 Freising-Weihenstephan, Germany; 7 BIOSS Centre for Biological Signalling Studies, University of Freiburg, 79104 Freiburg, Germany; 8 University Hospital Zurich, Institute of Surgical Pathology, 8091 Zurich, Switzerland; 9 Technische Universität München, ZIEL—Institute for Food & Health, 85350 Freising-Weihenstephan, Germany; 10 Technische Universität München, Department of Surgery, 81675 Munich, Germany; 11 German Cancer Research Center (DKFZ), Division of Chronic Inflammation and Cancer, 69120 Heidelberg, Germany

## Abstract

Control of intestinal epithelial stemness is crucial for tissue homeostasis. Disturbances in epithelial function are implicated in inflammatory and neoplastic diseases of the gastrointestinal tract. Here we report that mitochondrial function plays a critical role in maintaining intestinal stemness and homeostasis. Using intestinal epithelial cell (IEC)-specific mouse models, we show that loss of HSP60, a mitochondrial chaperone, activates the mitochondrial unfolded protein response (MT-UPR) and results in mitochondrial dysfunction. HSP60-deficient crypts display loss of stemness and cell proliferation, accompanied by epithelial release of WNT10A and RSPO1. Sporadic failure of Cre-mediated *Hsp60* deletion gives rise to hyperproliferative crypt foci originating from OLFM4^+^ stem cells. These effects are independent of the MT-UPR-associated transcription factor CHOP. In conclusion, compensatory hyperproliferation of HSP60^+^ escaper stem cells suggests paracrine release of WNT-related factors from HSP60-deficient, functionally impaired IEC to be pivotal in the control of the proliferative capacity of the stem cell niche.

## Abstract (web summary)

It is unclear what role mitochondrial function plays in maintaining intestinal epithelial cell (IEC) homeostasis. Here, the authors deplete a mitochondrial chaperone, heat shock protein 60 (HSP60) in IEC and observe a loss of stemness and cell proliferation, and suggest this is accompanied by a compensatory release of WNT-related factors.

## Graphical abstract

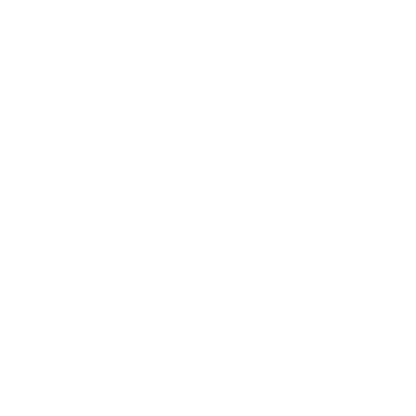

The intestinal epithelial cell (IEC) layer constitutes a rapidly self-renewing interface in intimate contact with the enteral environment and the immune system of the host, enabling intestinal homeostasis. Disturbances of this homeostasis can give rise to chronic degenerative diseases of the gastrointestinal tract such as colorectal cancer (CRC) or inflammatory bowel diseases (IBD)^1^. Genome-wide association studies on >25,000 IBD patients comprising Crohn's disease (CD) and ulcerative colitis (UC) identified >200 susceptibility loci associated with IBD^2^,^3^ and about 20 loci associated with CRC^4^. Many of the so far investigated genes affect the functions of the intestinal epithelium^5^,^6^.

Epithelial crypts are the sites where epithelial cells differentiate from pluripotent stem cells. After several cycles of proliferation in the transit amplifying zone, stem-cell-derived progenitor cells differentiate into absorptive enterocytes or into cells of the secretory lineage (goblet, enteroendocrine and tuft cells)^7^. On the contrary, Paneth cells directly descend from stem cells and remain within the crypt to fulfil their role in antimicrobial defence and stem cell maintenance^8^,^9^. Defects in epithelial cell homeostasis affecting antimicrobial defence, barrier permeability and IEC-immune cell interaction are crucial features of disease pathogenesis of IBD^5^. Chronic inflammation is a major risk factor for the development of CRC, largely accounting for the increased risk seen in IBD patients^10^. Specifically for CRC many of the so far identified loci have been associated with the regulation of proliferation^4^.

To maintain homeostasis and IEC functionality on a cellular level, the abundance and capacity of organelles need to be tightly regulated and adapted to the actual cellular demand. One critical process that limits organelle and cellular function is the availability of properly folded and functional proteins. Unfolded protein responses (UPR) are autoregulatory mechanisms that evolved in the cytoplasm, the endoplasmic reticulum (ER) and mitochondria to ensure adaptation to fluctuating cellular demands of proteins upon environmental triggers and/or host-derived signals^11^,^12^,^13^. Triggers affecting protein homeostasis comprise infections, oxidative stress and metabolic alterations^14^,^15^. UPR of the ER is particularly important for Paneth and Goblet cell function, since these cells are specialized in the production and secretion of proteins assembled in the ER. We and others provided evidence that a deregulated ER-UPR in IEC is indeed relevant for the pathogenesis in human IBD^16^,^17^,^18^. Furthermore, recent studies revealed that an activated ER-UPR in crypt base columnar cells via stem cell-specific depletion of the ER chaperone glucose-regulated protein 78 (GRP78) antagonizes stem cell properties and proliferation^19^. Besides in the ER, UPR mechanisms also evolved in mitochondria (MT-UPR), and an adequate amount of properly folded and functional proteins is essential for their fundamental metabolic functions (for example, oxidative phosphorylation and beta oxidation)^20^. Consistently, Mohrin *et al*.^21^ showed that a Sirtuin 7-mediated branch of the MT-UPR couples cellular energy metabolism and proliferation in hematopoietic stem cells. ER-UPR and MT-UPR signalling share certain features such as activation of the transcription factor C/EBP homologous protein (CHOP), and consistently, epithelial mitochondrial dysfunction and failure in cellular oxidative metabolism have been implicated in the pathogenesis of IBD^22^ and CRC^6^.

Studies in yeast from the late 1980s attribute an essential role to heat shock protein 60 (HSP60) in mitochondrial protein homeostasis^23^,^24^. Mutations in the *HSPD1* gene encoding HSP60 were discovered to cause hereditary spastic paraplegia in humans, a severe neurodegenerative disorder caused by mitochondrial dysfunction^25^,^26^,^27^. Moreover, constitutive HSP60 deficiency antagonizes cell viability in yeast^28^ and leads to embryonic lethality in mice^29^. We demonstrated increased HSP60 expression and activated MT-UPR signalling in the epithelium of IBD patients as well as murine models of colitis and proposed a link between ER- and MT-UPR through the cytoplasmic kinase PKR^30^. MT-UPR in mammals is rather poorly described, but mechanistic studies in a primate-derived cell line identified the transcription factor CHOP and its cofactor C/EBPβ to induce expression of MT-UPR responsive chaperones like HSP60, its co-chaperone HSP10 and proteases like ATP-dependent caseinolytic peptidase proteolytic subunit homologue (CLPP)^31^,^32^,^33^. By using an epithelial-specific *Chop* transgenic mouse model, we recently showed delayed epithelial proliferation and intestinal wound healing in response to increased levels of CHOP, suggesting CHOP to affect intestinal homeostasis by attenuating cell cycle progression^34^.

To investigate the role of the mitochondrial chaperone HSP60 in the regulation of epithelial cell homeostasis in the intestine, we generated epithelial-specific knockout mice. In the present study we show that HSP60 deficiency leads to mitochondrial dysfunction and antagonizes epithelial stem cell homeostasis through CHOP-independent mechanisms. The mitochondrial dysfunction was associated with paracrine release of WNT-related signals and hyperproliferation of residual stem cells that have escaped *Hsp60* deletion. Conclusively, these data indicate a fundamental role of mitochondrial function in the control of the epithelial stem cell niche.

## Results

### Epithelial *Hsp60* deficiency impacts IEC proliferation

**Figure 1:**
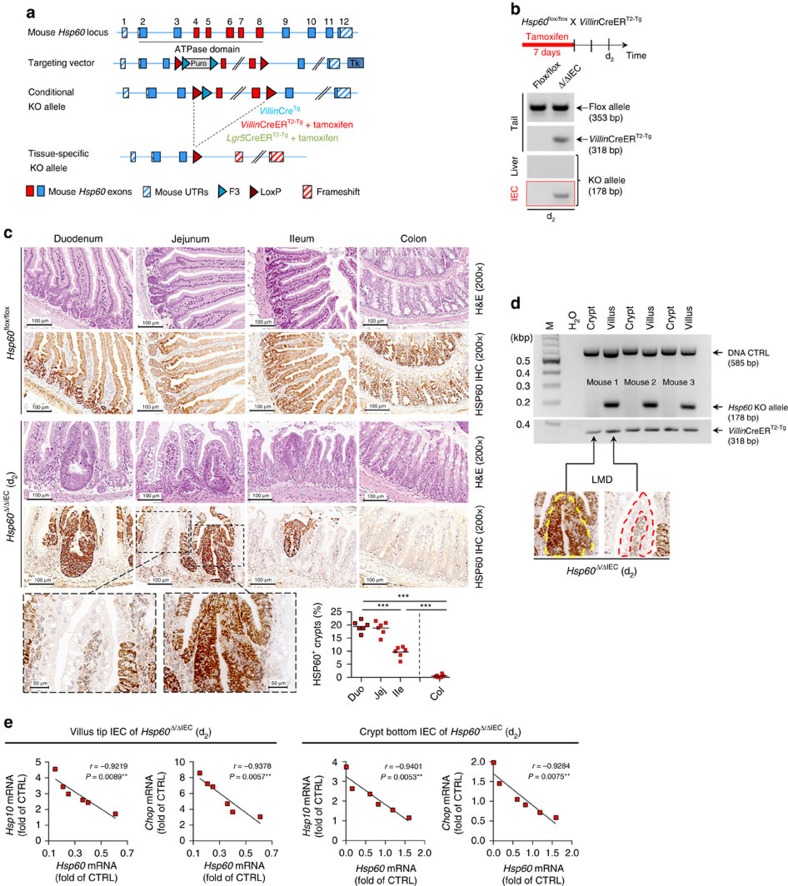
Formation of hyperproliferative crypt nodules in the small intestine of *Hsp60*^Δ/ΔIEC^ mice. (**a**) Schematic illustration of the targeted genomic modifications to generate mice carrying a conditional knockout allele for *Hsp60* (Flox allele). Expression of Cre recombinase and induction of Cre activity by tamoxifen generate the Hsp60 knockout specifically in intestinal epithelial cells (IEC) or intestinal stem cells (ISC). (**b**) Schedule for oral tamoxifen administration to induce HSP60 deficiency in IEC of adult mice. Agarose gels showing the presence of the knockout allele specifically in IEC isolates. (**c**) Representative H&E and corresponding HSP60 IHC stainings of *Hsp60*^flox/flox^ and *Hsp60*^Δ/ΔIEC^ mice along the intestinal tract. Images of HSP60 IHC in higher magnification show HSP60-deficient villus versus HSP60-positive crypt regions of the jejunum. HSP60-positive crypt nodules in *Hsp60*^Δ/ΔIEC^ mice were counted along the intestinal tract using HSP60 IHC stainings. The graph represents quantifications of *Hsp60*^Δ/ΔIEC^ mice (*N*=6) with >100 crypts counted per animal. Lines indicate mean numbers. One-way analysis of variance (ANOVA) followed by Dunn's test was used to test for significance. (**d**) Genomic DNA was isolated from villi and hyperproliferative crypt nodules of *Hsp60*^Δ/ΔIEC^ mice using laser-dissection microscopy (*N*=3). Presence and absence of the *Hsp60* knockout allele and Cre transgene was determined via PCR. DNA control PCR were run to check for equal loading (**e**) Correlation (Pearson) of MT-UPR marker gene expression with *Hsp60* mRNA levels in IEC isolated from jejunal fractions of villus tip and crypt bottom of *Hsp60*^Δ/ΔIEC^ mice (*N*=6). *P* values indicate one-sided significance. Asterisks indicate significant differences *P<0.05, **P<0.01, ***P<0.001; NS, not significant.

To investigate the role of HSP60 in IEC homeostasis, we generated conditional knockout mice. Exons 4–8 covering main parts of the ATPase domain were flanked with loxP sites via homologous recombination in embryonic stem cells. Tissue-specific deletion of the conditional knockout allele (*Hsp60*^flox/flox^) was achieved by breeding mice to different Cre recombinase expressing mice (Fig. 1a). Crossing of *Hsp60*^flox/flox^ mice with *Villin*Cre transgenic mice caused embryonic lethality due to general developmental retardation shortly after the onset of *Villin* expression (Supplementary Fig. 1)^35^. To circumvent this problem we used tamoxifen-inducible *Villin*CreER^T2-Tg^ mice to generate an epithelial-specific *Hsp60* knockout in adult mice (Fig. 1b). Due to severe wasting mice fulfilled abortion criteria and were killed 2 days after the end of tamoxifen administration (d_2_). Histopathological evaluation of intestinal tissue sections in *Hsp60*^Δ/ΔIEC^ mice revealed numerous hyperproliferative crypt foci forming nodular structures with decreasing abundance from the duodenum to the colon (Fig. 1c). Immunohistochemical stainings confirmed the loss of epithelial HSP60 protein expression in most intestinal tissue areas (Fig. 1c). In contrast, hyperproliferative crypt nodules displayed strong expression of HSP60 (Fig. 1c). Laser-dissection microscopy was used to isolate genomic DNA from the epithelium of HSP60-positive, hyperproliferative crypt nodules and HSP60-negative villus tips of jejunal tissue sections. Interestingly, and in contrast to the villus tip, *Hsp60* knockout alleles were absent in IEC from hyperproliferative crypt nodules, indicating the presence of escaper regions for genomic recombination (Fig. 1d). HSP60 deficiency and the consequential disturbances in epithelial morphology were associated with low levels of inflammatory tissue activation, including moderately elevated numbers of F4/80-positive macrophages. Yet, expression levels of selected cytokines and chemokines showed no clear inflammatory pattern (Supplementary Fig. 2).

Investigating the impact of HSP60 loss on mitochondrial morphology, electron microscopic analysis was employed and indicated preservation of mitochondrial numbers and diameter in the epithelium of *Hsp60*^Δ/ΔIEC^ mice. However, mitochondrial cristae appeared less structured under conditions of HSP60 deficiency (Supplementary Fig. 3A), suggesting altered mitochondrial functionality.

### Loss of HSP60 activates MT-UPR independently of CHOP

To characterize the impact of HSP60 deficiency on UPR activation in the epithelium, we differentially isolated jejunal IEC along the villus–crypt axis. Isolated IEC from the villus tip compartment were characterized by a high degree of HSP60 deficiency, whereas the crypt bottom combined HSP60-deficient areas and HSP60-positive hyperproliferative crypt foci. In IEC from villus tip and crypt bottom, expression of the co-chaperone *Hsp10* and the transcription factor *Chop*, two surrogate markers of MT-UPR, highly correlated with *Hsp60* deficiency, confirming the presence of activated MT-UPR in both compartments (Fig. 1e).

Since CHOP is involved in the induction of apoptotic processes^36^,^37^, we performed a TUNEL assay to investigate whether the induction of *Chop* expression seen in *Hsp60*^Δ/ΔIEC^ led to increased apoptosis of IEC. However, the lack of TUNEL-positive epithelial cells clearly indicated the absence of pro-apoptotic mechanisms in the entire epithelium including highly *Chop*-expressing villi IEC. (Supplementary Fig. 3B).

**Figure 2:**
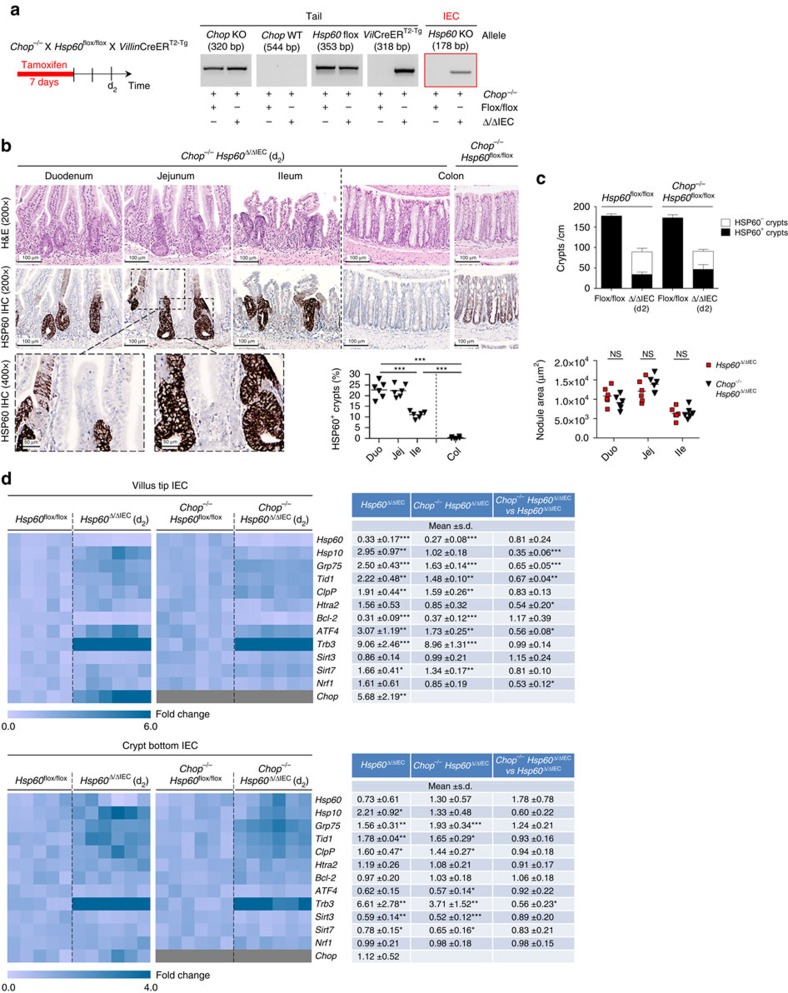
Formation of hyperproliferative crypt nodules and MT-UPR activation is independent of CHOP-mediated signalling. (**a**) Schedule for oral tamoxifen administration to induce HSP60 deficiency in IEC of adult *Chop*^−/−^ *Hsp60*^Δ/ΔIEC^ mice. Agarose gels showing the presence of the knockout allele specifically in IEC isolates. (**b**) Representative H&E and corresponding HSP60 IHC stainings of *Chop*^−/−^ *Hsp60*^Δ/ΔIEC^ mice along the intestinal tract. Images of HSP60 IHC in higher magnification show HSP60-deficient villus versus HSP60-positive crypt regions of the jejunum. HSP60-positive crypt nodules in *Chop*^−/−^ *Hsp60*^Δ/ΔIEC^ mice were counted along the intestinal tract using HSP60 IHC stainings. The graph represents quantifications of *Hsp60*^Δ/ΔIEC^ mice (*N*=6) with >100 crypts counted per animal. Lines indicate mean numbers. One-way analysis of variance (ANOVA) followed by Dunn's test was used to test for significance. (**c**) Quantification of HSP60-positive and HSP60-negative crypt numbers and total area of HSP60-positive crypt nodules of *Hsp60*^Δ/ΔIEC^ and *Chop*^−/−^ *Hsp60*^Δ/ΔIEC^ mice revealed no differences between genotypes (*N*=6); unpaired *t*-tests. Bars represent means+s.e.m. Lines in the dot plot indicate mean numbers. (**d**) qRT–PCR analysis of MT-UPR-associated genes in villus (upper panel) and crypt (low panel) IEC isolated from *Hsp60*^Δ/ΔIEC^ (*N*=6) and *Chop*^−/−^ *Hsp60*^Δ/ΔIEC^ mice (*N*=6) and corresponding controls (*N*=5 and *N*=6). Statistical analysis was performed using unpaired *t*-tests. Asterisks indicate significant differences *P<0.05, **P<0.01, ***P<0.001; NS, not significant.

To test whether the presence of HSP60-positive hyperproliferative crypt foci might be due to CHOP-mediated signals of the surrounding HSP60-deficient epithelium, we next generated *Chop*^−/−^ *Hsp60*^Δ/ΔIEC^ mice. Interestingly, the lack of CHOP did not interfere with formation and extent of HSP60-positive hyperproliferative crypt nodules (Fig. 2a–c; Supplementary Fig. 4). However, the absence of severe wasting in *Chop*^−/−^ *Hsp60*^Δ/ΔIEC^ mice allowed for tissue analysis beyond time point d_2_, demonstrating complete tissue reconstitution. Neither hyperproliferative crypt foci forming nodular structures nor increased expression of the proliferation marker Ki67 were detectable 12 days after the end of tamoxifen administration (d_12_). Already by d_8_, HSP60-deficient crypts were completely resorbed with the total number of (HSP60-positive) crypts per centimetre again reaching control levels (Supplementary Fig. 5A,B), indicating formation of hyperproliferative crypt nodules to be an essential step in epithelial reconstitution. Further analysis of MT-UPR-associated gene expression indicated activated MT-UPR signalling in villus tip as well as crypt bottom IEC from both *Hsp60*^Δ/ΔIEC^ and *Chop*^−/−^ *Hsp60*^Δ/ΔIEC^ mice, with only minor differences between the animal models (Fig. 2d). Thus, presence of hyperproliferative crypt foci and MT-UPR activation in response to epithelial HSP60 loss seemed to be independent of the MT-UPR-associated transcription factor CHOP.

**Figure 3:**
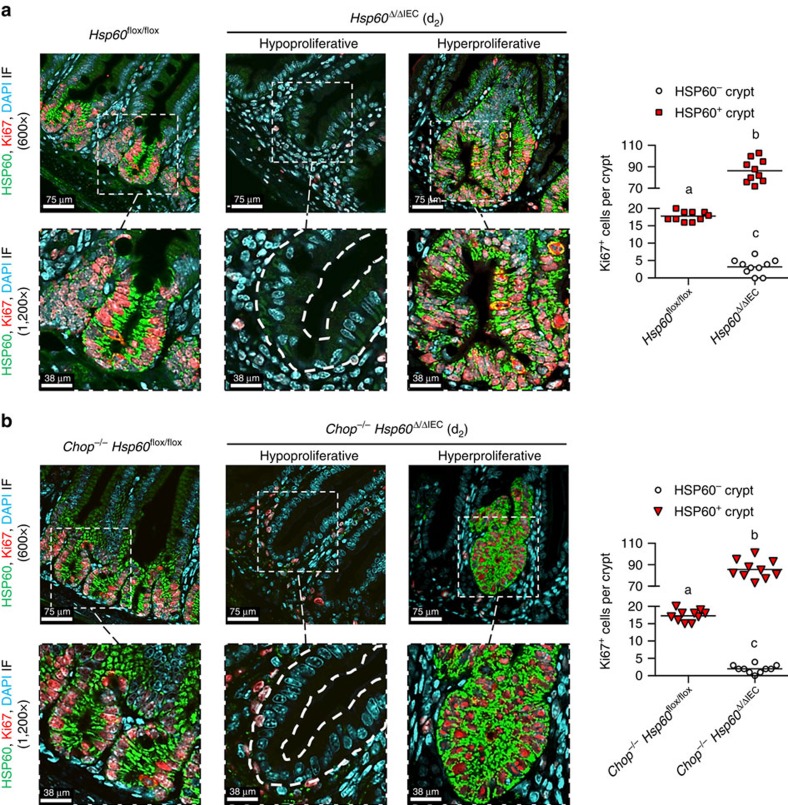
HSP60 levels determine the proliferative capacity of the crypt. (**a**) IF co-staining of HSP60 and Ki67 on jejunal sections including detailed images in higher magnification of *Hsp60*^flox/flox^ controls and *Hsp60*^Δ/ΔIEC^ mice (DAPI stains nuclei in cyan; dotted lines indicate localization of cryptal epithelium). Quantification of Ki67-positive cells in HSP60-positive and HSP60-negative crypts (*N*=5; 2 regions per mouse). (**b**) Parallel evaluation as in (**a**) for *Chop*^−/−^ *Hsp60*^Δ/ΔIEC^ mice and *Chop*^−/−^ *Hsp60*^flox/flox^ controls. Lines in the dot plots indicate mean numbers. (a,b,c), significantly different from each other. One-way analysis of variance (ANOVA) and appropriate *post-hoc* test were used for statistical analysis.

Dissecting the impact of HSP60 deficiency on IEC proliferation, immunofluorescence analysis was used to visualize HSP60 and Ki67 in paraffin-embedded jejunal tissue sections. In crypts of *Hsp60*^flox/lox^ (CTRL) mice, HSP60 co-localized with Ki67-positive cells. In contrast, Ki67 expression was largely absent in HSP60-deficient crypts of *Hsp60*^Δ/ΔIEC^ mice (Fig. 3a). Surrounded by this HSP60-deficient epithelial environment, HSP60 positive, hyperproliferative crypt nodules were strongly positive for Ki67 expression. The number of Ki67 positive cells in these nodules was three to four times higher compared with CTRL crypts (Fig. 3). This phenotype was confirmed in *Chop*^−/−^ *Hsp60*^Δ/ΔIEC^ mice (Fig. 3b), again supporting CHOP-independent mechanisms to induce hyperproliferative crypt foci.

### Loss of HSP60 results in mitochondrial dysfunction

**Figure 4:**
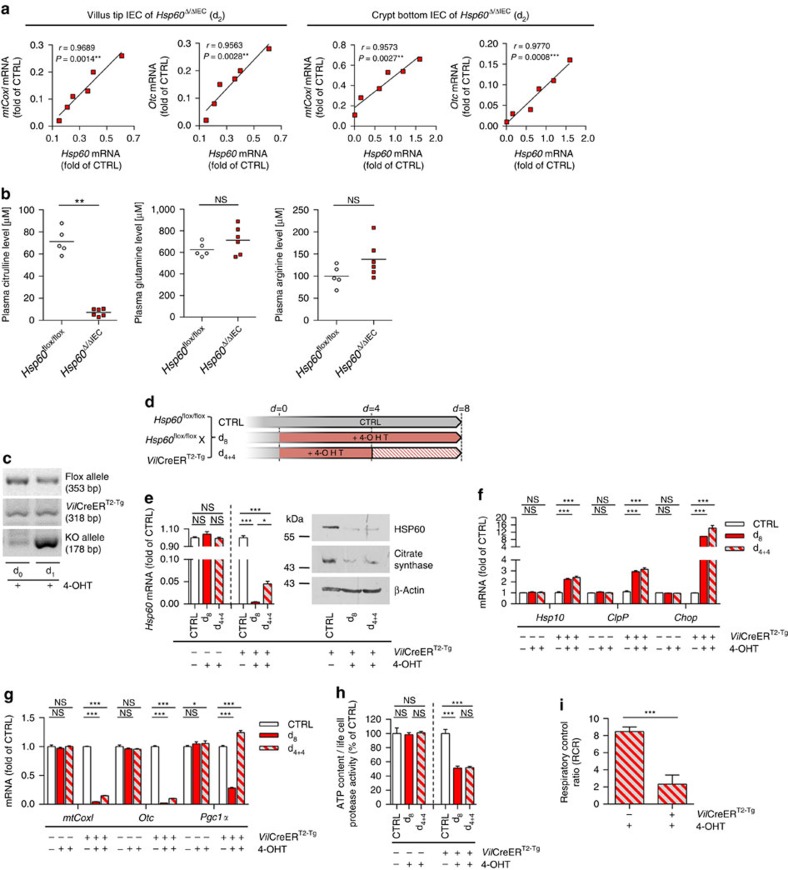
HSP60 deficiency impairs mitochondrial function. (**a**) Correlation (Pearson) of expression levels of *mtCoxI* and *Otc*, involved in mitochondrial function, with *Hsp60* mRNA levels in isolated IEC from villus (left) and crypt (right) compartment of *Hsp60*^Δ/ΔIEC^ mice (*N*=6 per genotype). *P* values indicate one-sided significance. (**b**) Plasma citrulline (left), glutamine (middle) and arginine (right) level in *Hsp60*^Δ/ΔIEC^ mice at d_2_ (*N*=6) and *Hsp60*^flox/flox^ controls (*N*=5). Statistical analysis was performed using unpaired *t*-tests. (**c**) Agarose gel showing the presence of the *Hsp60*-knockout allele in genomic DNA isolated from organoids at 1 day after tamoxifen (4-OHT) addition. (**d**) Schematic illustration of the experimental set-up using small intestinal organoids *ex vivo*. Organoids were isolated from *Hsp60*^flox/flox^, *Villin*CreER^T2-Tg^ and according *Villin*CreER^T2^ negative control mice and distributed to three protocols. One-way analysis of variance and appropriate *post hoc* test were used for statistical analysis. (**e**) mRNA (left) and protein (right) expression levels of *Hsp60* in organoids after 4-OHT treatment. Protein levels of mitochondria-located citrate synthase and β-Actin serving as loading control are also shown. (**f**) qRT–PCR analysis of target genes involved in MT-UPR. (**g**) qRT–PCR analysis of target genes involved in mitochondrial function. (**h**) ATP content of organoids relative to life cell protease activity measured by a fluorescence assay. (**i**) Respiratory capacity of mitochondria was measured by high-resolution respirometry. To exclude toxicity-related artefacts of 4-OHT, effects on CreER^T2-Tg^ organoids were directly compared to CreER^T2^-negative organoids both receiving 4-OHT. Statistics were performed by unpaired *t*-test. Data from organoid experiments derive from at least 3 independent experiments. Bars represent mean+s.e.m. Asterisks indicate significant differences *P<0.05, **P<0.01, ***P<0.001; NS, not significant.

In addition to UPR activation, HSP60 deficiency diminished expression of marker genes related to mitochondrial function, mitochondrial DNA-encoded *Cyclooxygenase I* (*mtCoxI*) and *Ornithine transcarbamylase* (*Otc*; Fig. 4a). Again demonstrating the CHOP-independency of the observed effects, *Chop*^−/−^ *Hsp60*^Δ/ΔIEC^ mice showed comparably reduced expression levels of both genes (Supplementary Fig. 5C). While MT-COXI is involved in oxidative phosphorylation, OTC is part of the urea cycle and syntheizes the amino-acid citrulline from glutamine in the gut. In kidneys, citrulline is converted into arginine^38^. Constituting a functional read-out of IEC mitochondrial function, *Hsp60*^Δ/ΔIEC^ mice displayed markedly reduced plasma citrulline concentrations with concomitantly unaltered levels of glutamine and arginine (Fig. 4b).

To further validate the effect of HSP60 loss on mitochondrial function, we used small intestinal organoid culture and induced *Hsp60* knockout *ex vivo*. Already after one day of 4-hydroxytamoxifen (4-OHT) treatment (d_1_), the knockout allele was detectable at the genomic level (Fig. 4c). Accordingly, mRNA expression of *Hsp60* was largely abolished at d_1_ (Supplementary Fig. 6A,B). In the following, organoids were treated with 4-OHT for 8 consecutive days (d_8_) or 4 days followed by 4 days of recovery (d_4+4_) (Fig. 4d). Hsp60 mRNA and protein expression were virtually absent at d_8_. Withdrawal of 4-OHT in the recovery phase lead to moderately elevated *Hsp60* mRNA levels compared with 8 days of continuous 4-OHT exposure, an effect that was not detectable on protein level (Fig. 4e). Levels of Citrate synthase, mirroring changes in HSP60 expression confirmed the profound reduction of mitochondrial matrix protein folding capacity, since this enzyme is known to be folded with assistance of HSP60 after import into the mitochondria^39^. qPCR analysis indicated the induction of MT-UPR marker genes *Hsp10*, *ClpP* and *Chop* upon *Hsp60* knockout, reflecting the effects seen *in vivo* (Fig. 4f).

Again confirming changes observed in *Hsp60* mice, mRNA expression of *mtCoxI* and *Otc* were abolished. Concomitantly, peroxisome proliferator-activated receptor coactivator 1 alpha (*Pgc1α*), a master regulator of mitochondrial biosynthesis, showed a significant decrease on mRNA level at d_8_ (Fig. 4g). Interestingly, mRNA level of *Pgc1α* were elevated in the d_4+4_ approach, suggesting enhanced mitogenesis as an attempted to restore epithelial homeostasis. Demonstrating mitochondrial dysfunction induced by epithelial HSP60 loss, cellular ATP content was significantly reduced (Fig. 4h). Functionally most important, the respiratory control ratio (RCR) was markedly diminished indicating attenuated coupling of respiratory chain proton pump activities and ATP synthesis (Fig. 4i). These findings support the hypothesis of reduced mitochondrial function.

### Loss of HSP60 impacts epithelial stemness

**Figure 5:**
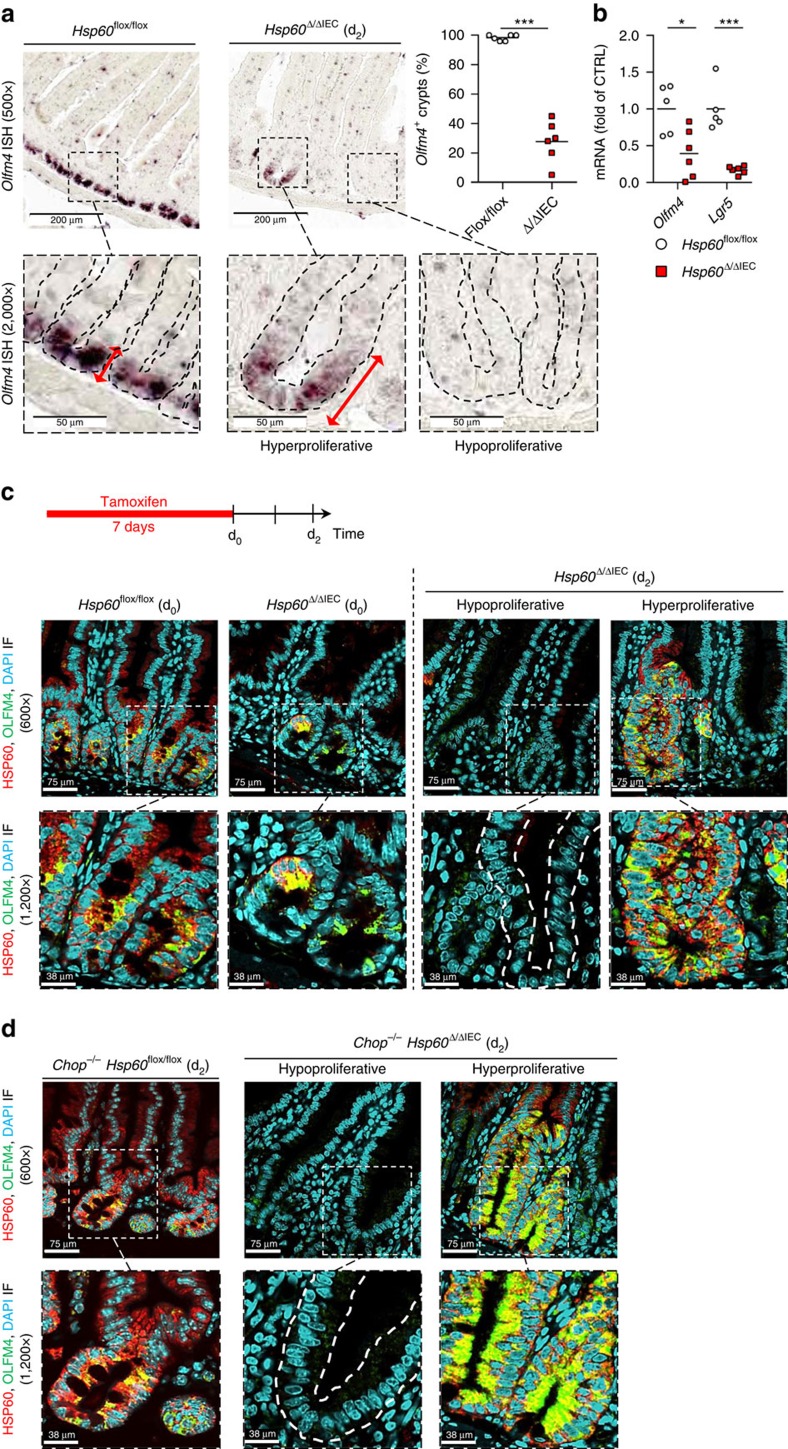
HSP60 loss antagonizes stemness while HSP60-positive escaper stem cells hyperproliferate. (**a**) Images showing representative *in situ* hybridizations for *Olfm4* mRNA on jejunal sections at d_2_ including detailed images in higher magnification and quantification of *Olfm4*-positive crypts (*N*=6); dotted lines indicate localization of cryptal epithelium; red arrows indicate zones of *Olfm4* expression). The dot plot indicates number of *Olfm4*^+^ crypts in *Hsp60*^flox/flox^ and *Hsp60*^Δ/ΔIEC^ mice at d_2_. (**b**) qRT–PCR analysis of stem cell markers Olfm4 and Lgr5 was performed on isolated crypt bottom IEC of *Hsp60*^flox/flox^ (*N*=5) versus *Hsp60*^Δ/ΔIEC^ mice (*N*=6). Lines in the dot plots indicate mean numbers. All statistical analyses were performed via unpaired *t*-tests comparing genotypes. (**c**) Schedule for oral tamoxifen administration to induce HSP60 deficiency in IEC. IF images show HSP60 and OLFM4 expression at two different time points (d_0_ and d_2_) in jejunal sections of *Hsp60*^Δ/ΔIEC^ and *Hsp60*^flox/flox^ mice (DAPI stains nuclei in cyan). Representative pictures of *N*=5 per genotype. (**d**) Parallel analysis as in **c** for *Chop*^−/−^ *Hsp60*^Δ/ΔIEC^ mice and corresponding *Chop*^−/−^ *Hsp60*^flox/flox^ controls. Asterisks indicate significant differences *P<0.05, **P<0.01, ***P<0.001; NS, not significant.

**Figure 6:**
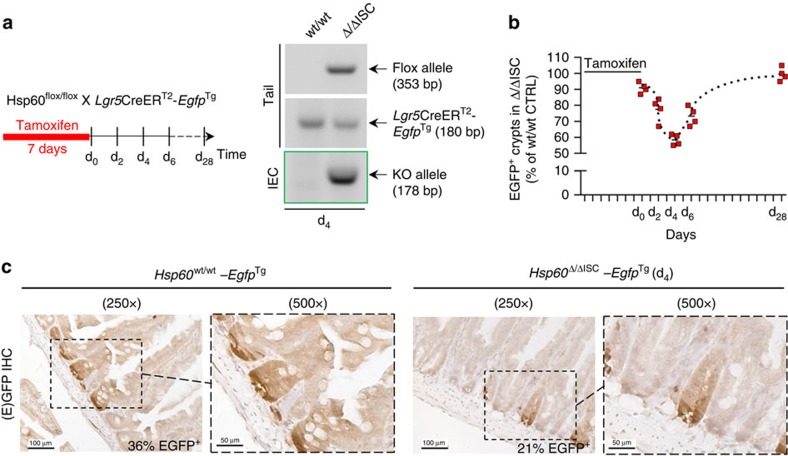
HSP60 deficiency in Lgr5-positive cells induces intestinal stem cell loss. (**a**) Schedule for oral tamoxifen administration to induce HSP60 deficiency in intestinal stem cells (ISC, left) in *Hsp60*^*flox*/flox^, *Lgr5*CreER^T2^-*Egfp*^Tg^ mice. Agarose gel validating the presence of the knockout allele in IEC isolates (middle). (**b**) Quantification of EGFP-positive, *Lgr5* expressing ISC containing crypts at different time points after ISC-specific *Hsp60* deletion (*N*=4 per genotype and time point) (right). (**c**) Representative IHC stainings for EGFP at d_4_ are shown and the corresponding percentages of EGFP-positive crypts are indicated (lower panel).

To determine the cellular origin of HSP60-positive, hyperproliferative crypt nodules, *in situ* hybridization was performed for the stem cell marker Olfactomedin 4 (*Olfm4*). While expression of *Olfm4* was not detectable in morphologically normal jejunal crypts of *Hsp60*^Δ/ΔIEC^ mice, hyperproliferative crypt nodules showed enlarged regions of *Olfm4*-positive cells (Fig. 5a). Decreased transcript levels of *Olfm4* in total IEC were paralleled by strongly reduced *Lgr5* mRNA expression (Fig. 5b). To investigate early events in the development of hyperproliferative crypt nodules, *Hsp60*^Δ/ΔIEC^ mice were analysed directly after the end of tamoxifen administration (d_0_). At this time point, only few escaper cells double-positive for HSP60 and OLFM4 were detected in scattered crypts indicating that hyperproliferative crypt nodules seen at d_2_ originated from HSP60- and OLFM4-double-positive stem cells (Fig. 5c). Conversely, HSP60-deficient, OLFM4-positive stem cells still visible at d_0_ disappeared until d_2_, resulting in hypoproliferative crypts lacking stem as well as transit amplifying cells (Figs 5c and 3a). Thus, HSP60 depletion in IEC not only seems to antagonize proliferation but also stemness in the crypt compartment. Contrarily, extensive growth of stem cells sporadically escaping target gene deletion, suggested Hsp60 and mitochondrial function also to be involved in the positive control of stem cell proliferation. According to the previous results (Figs 1, 2, 3), both loss and gain of stem cell proliferation were independent of CHOP, as demonstrated by *Chop*^−/−^ *Hsp60*^Δ/ΔIEC^ mice (Fig. 5d). Directly addressing the role of HSP60 and mitochondrial function on stem cell homeostasis, we bred *Hsp60*^flox/flox^ mice to *Lgr5*CreER^T2-Tg^ mice, generating a stem cell-specific HSP60-deficient mouse model (*Hsp60*^Δ/ΔISC^). Using the *Lgr5-EGFP-ires-CreERT2* knock-in mouse, in which expression of green fluorescent protein (eGFP) is driven from the *Lgr5* locus allowed following the fate of Lgr5-positive stem cells after *Hsp60* knockout. A time-dependent analysis of EGFP-positive cell numbers revealed a transient drop in stem cell numbers reaching a minimum 4 days (d_4_) after the end of tamoxifen administration. A rapid regain of Lgr5-positive, EGFP expressing stem cells was already detectable 2 days later (d_6_), resulting in a complete regeneration and return to control cell numbers at d_28_ (Fig. 6). Hyperproliferative crypt nodules were not observed at any time in *Hsp60*^Δ/ΔISC^ mice, suggesting HSP60 loss in stem cells not to be sufficient to induce hyperproliferation, but to be dependent on paracrine signals arising from HSP60-deficient IEC.

### Stem cell hyperproliferation is dependent on WNT signalling

**Figure 7:**
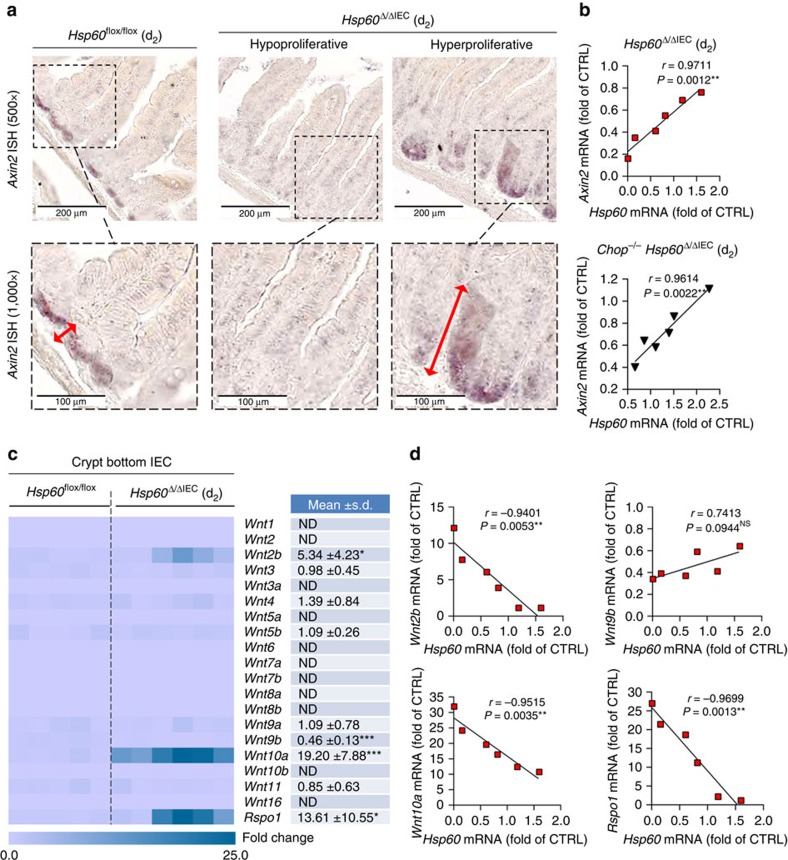
Stem cell hyperproliferation is associated with epithelial induction of WNT-related signals. (**a**) Representative pictures of *in situ* hybridization for WNT-target gene *Axin2* mRNA in jejunal sections at d_2_ including detailed images in higher magnification (red arrows indicate zones of *Axin2* expression) (**b**) Correlation analysis (Pearson) of *Axin2* and *Hsp60* mRNA expression levels in IEC isolated from the crypt of *Hsp60*^Δ/ΔIEC^ (upper graph) and *Chop*^−/−^ *Hsp60*^Δ/ΔIEC^ (lower graph) mice (*N*=6). *P* values indicate one-sided significance. (**c**) qRT–PCR analysis of WNT ligands including the WNT enhancer *Rspo1* in IEC isolated from the crypt bottom of *Hsp60*^Δ/ΔIEC^ mice (*N*=6) versus *Hsp60*^*flox*/flox^ control mice (*N*=5). Statistical analyses were performed using unpaired *t*-tests. (**d**) Correlation analysis (Pearson) of significantly regulated WNT factors with *Hsp60* levels in *Hsp60*^Δ/ΔIEC^ mice (*N*=6). *P* values indicate one-sided significance. Asterisks indicate significant differences *P<0.05, **P<0.01, ***P<0.001; NS, not significant; ND, not detectable.

**Figure 8:**
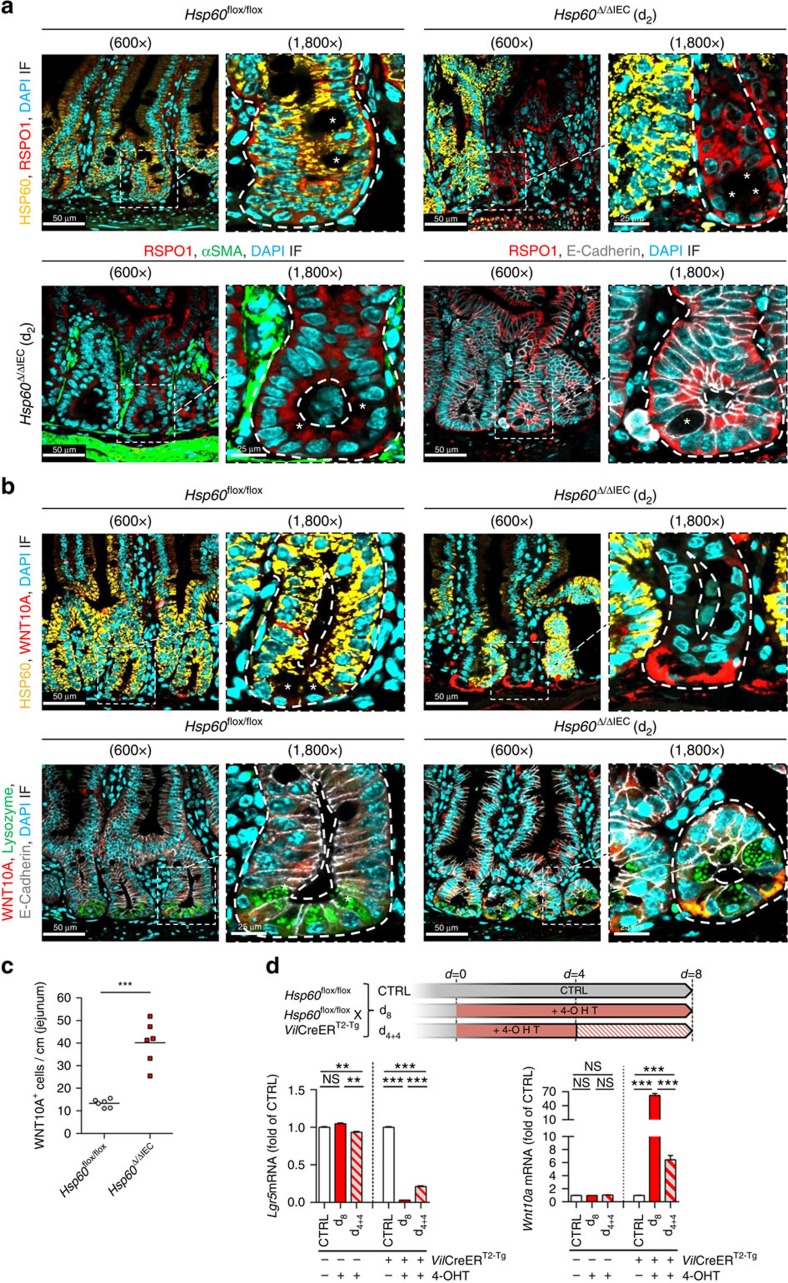
HSP60 loss is associated with epithelial induction of WNT-related signals. (**a**) IF images show epithelial RSPO1 (red) and HSP60 (yellow) expression at d_2_ in jejunal sections from *Hsp60*^Δ/ΔIEC^ and *Hsp60*^*flox*/flox^ mice. E-Cadherin (grey) and αSMA (green) were used as epithelial and mesenchymal cell marker, respectively (DAPI stains nuclei in cyan, asterisks indicate Paneth cells). (**b**) IF images show epithelial WNT10A (red) and HSP60 (yellow) expression at d_2_ in jejunal sections from *Hsp60*^Δ/ΔIEC^ and *Hsp60*^*flox*/flox^ mice. Lysozyme (green) and E-Cadherin (grey) were used as Paneth cell and epithelial cell marker, respectively (DAPI stains nuclei in cyan). WNT10A and Lysozyme co-stained (yellow, lower panel). (**c**) Quantification of WNT10A-positive cells in the jejunum of *Hsp60*^Δ/ΔIEC^ and *Hsp60*^*flox*/flox^ mice (*N*=6). Lines in the dot plot indicate mean numbers. Statistical analysis was performed using unpaired *t*-test. (**d**) Experimental scheme to induce *Hsp60* loss in small intestinal organoids. qRT–PCR analysis of *Lgr5* and *Wnt10a* mRNA expression in organoids following gene knockout. Data from organoid experiments derive from at least 3 independent experiments. Bars represent mean +s.e.m. One-way analysis of variance and appropriate *post-hoc* tests were used for statistical analysis. Asterisks in **c**, **d** indicate significant differences *P<0.05, **P<0.01, ***P<0.001; NS, not significant.

WNT signalling is crucial for stemness and proliferation in the intestinal crypt. *In situ* hybridization revealed loss of *Axin2* expression, a target gene of WNT signalling, in jejunal crypts of *Hsp60*^Δ/ΔIEC^ mice, with the exception of hyperproliferative crypt nodules where the expression zone was enlarged (Fig. 7a). Quantitative Real-time PCR (qRT–PCR) analysis confirmed a positive correlation of *Axin2* and *Hsp60* expression levels in *Hsp60*^Δ/ΔIEC^ and *Chop*^−/−^ *Hsp60*^Δ/ΔIEC^ mice, supporting the hypothesis that proliferative control is independent of CHOP signalling (Fig. 7b). Expression analysis of growth factors related to the WNT family in crypt IEC revealed significantly altered mRNA levels for *Wnt2b*, *Wnt9b, Wnt10a* and *R-spondin 1* (*Rspo1*; Fig. 7c). Increased expression levels of *Wnt2b*, *Wnt10a* and the WNT-signalling enhancer *Rspo1* highly correlated with HSP60 deficiency (Fig. 7d, Supplementary Fig. 7A). Immunofluorescence analysis of RSPO1 and WNT10A, showing the most pronounced induction on mRNA level, confirmed RSPO1 expression in HSP60-deficient crypt IEC (Fig. 8a, Supplementary Fig. 7B,C) and indicated HSP60-negative Paneth cells as source of WNT10A (Fig. 8b). Staining for αSMA clearly demonstrated IEC but not tissue resident fibroblasts to be the major source of enhanced R-spondin 1 expression (Fig. 8a). The total number of WNT10A-positive IEC was significantly increased in the jejunum of *Hsp60*^Δ/ΔIEC^ mice (Fig. 8c). In addition, mRNA analysis of HSP60-deficient villus IEC showed induction of distinct Wnt-related growth factors, *Wnt4* and *Wnt11* (Supplementary Fig. 7D, Supplementary Fig. 8) most probably contributing to a pro-proliferative environment.

Reflecting the findings in *Hsp60*^Δ/ΔIEC^ mice, expression of the stem cell marker *Lgr5* was abolished in small intestinal organoids after *Hsp60* knockout (d_8_; Fig. 8d, Supplementary Fig. 6C). Allowing organoids to recover for 4 days in the absence of 4-OHT (d_4+4_) indicated a regain of *Lgr5* expression, clearly demonstrating the dynamic regulation of stemness. Concomitantly, an induction of *Wnt10a* expression was observed, most probably constituting an attempted to compensate the lack of epithelial renewal caused by HSP60 deficiency (Fig. 8d).

### WNT10A and RSPO1 rescue growth of *Hsp60* KO organoids

**Figure 9:**
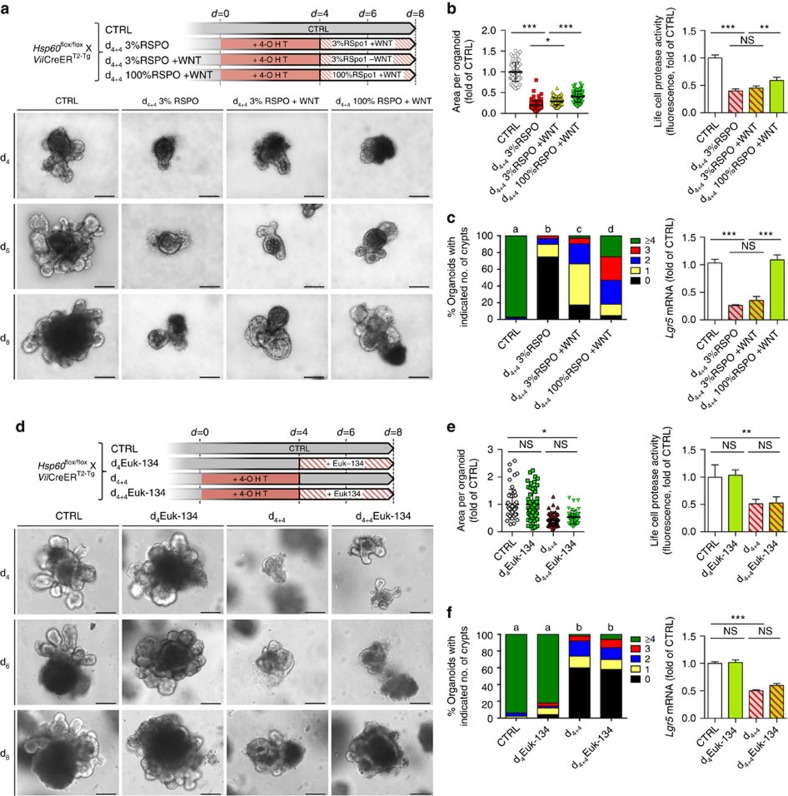
WNT10A and RSPO1 but not ROS scavenging rescues intestinal organoid growth after *Hsp60* knockout. Organoids were isolated from *Hsp60*^flox/flox^, *Villin*CreER^T2-Tg^ mice and distributed to four protocols. (**a**) Experimental scheme to show the effects of RSPO1 and WNT10A supplementation (100 ng ml^−1^) on Hsp60-deficient small intestinal organoids. Lower panel: representative pictures of the indicated treatments and time points. (**b**) Measurement of organoid area (left; *N*>60 per treatment) and life cell protease activity measured by fluorescence (right) following indicated treatments. (**c**) Quantification of *de novo* crypt formation (left); (a–d) significantly different from each other, Kruskal–Wallis test on ranks followed by Dunn's test. Right: qRT–PCR analysis of *Lgr5* mRNA expression in organoids in response to WNT10A treatment. (**d**) Experimental scheme to show the effects of the ROS scavenger Euk-134 (100 μM) on Hsp60-deficient small intestinal organoids. Lower panel: representative pictures of the indicated treatments and time points. (**e**) Organoid area (left; *N*>60 per treatment) and life cell protease activity measured by fluorescence (right) following indicated treatments. (**f**) Quantification of *de novo* crypt formation (left); (a,b), significantly different from each other, Kruskal–Wallis test on ranks followed by Dunn's test. Right: qRT–PCR analysis of *Lgr5* mRNA expression in organoids in response to Euk-134 treatment. Bars represent mean+s.e.m. Asterisks indicate significant differences *P<0.05; **P<0.01; ***P<0.001; NS, not significant. Unless otherwise indicated, one-way analysis of variance and appropriate *post hoc* tests were used for all statistical analyses. Data from organoid experiments derive from at least three independent experiments. Scale bars, 200μm.

Since the intestinal organoid culture system depends on R-spondin 1 supplementation in the medium to maintain growth and survival of organoids^40^, we first tested the impact of WNT10A beyond endogenous production on organoid growth with RSPO1 included in the medium in optimal concentration (1 μg ml^−1^/100%). The organoid culture medium was supplemented with WNT10A for 4 days after induction of *Hsp60* knockout by 4 days of treatment with 4-OHT. In HSP60-deficient organoids, WNT10A was able to partly rescue the proliferative stop/growth retardation induced by HSP60 loss, most likely by promoting growth of escaper cells as indicated by elevated *Hsp60* mRNA level (Supplementary Fig. 9). This effect was demonstrated by a significant increase in organoid area and *de novo* crypt formation as well as in the number of living cells measured by live-cell protease activity (Supplementary Fig. 9A–C). Accompanying growth enhancement, WNT10A treatment restored *Lgr5* mRNA expression to control levels (Supplementary Fig. 9C), indicating stem cell regain. In contrast, WNT10A treatment of *Hsp60*^flox/flox^ control organoids did not lead to a detectable growth improvement even though slightly elevated *Lgr5* expression levels were measured (Supplementary Fig. 9). This might be attributable to the organoid culture system, in which the abundance of growth factors in the medium probably exceeds the demand to maintain epithelial stemness, therefore, not constituting the limiting factor of growth under non-stressed conditions. To test the combined effect of the two most prominently regulated WNT-associated factors, RSPO1 and WNT10A, we decreased the concentration of RSPO1 in the culture medium to 3% (30 ng ml^−1^) of the optimal concentration after induction of *Hsp60* knockout. Addition of WNT10A under these conditions induced a slight enhancement of organoid growth measured by organoid area and *de novo* crypt formation. Consistently, optimal RSPO1 concentration (100%) and WNT10A supplementation for 4 days showed an additive effect leading to increased organoid area, *de novo* crypt formation, number of living cells, and restored *Lgr5* mRNA expression (Fig. 9a–c). Production of reactive oxygen species (ROS) is a hallmark of mitochondrial dysfunction and at the same time, ROS are thought to be important mediators of cellular stress signalling. In *Hsp60*^Δ/ΔIEC^ mice, immunohistochemical staining of 8-OHdG, a critical biomarker of oxidative stress and carcinogenesis^41^, did not provide evidence for increased oxidative stress in HSP60-deficient IEC (Supplementary Fig. 10A). Yet, mRNA expression analysis of genes involved in antioxidative response, *Hif1α*, *Ho1*, *Cat* and *Sod2* was not conclusive (Supplementary Fig. 10B). Therefore, we tested the properties of the ROS scavenger Euk-134 in the organoid culture system. No differences in organoid growth, *de novo* crypt formation, and *Lgr5* expression levels, neither in *Hsp60*^flox/flox^ control organoids nor in HSP60-deficient *Hsp60*^Δ/ΔIEC^ organoids, were observed after addition of Euk-134 (Fig. 9d–f). Hence, mitochondrial ROS production caused by HSP60 loss-associated mitochondrial dysfunction seems to play a minor role in the control of epithelial proliferation and stemness.

**Figure 10:**
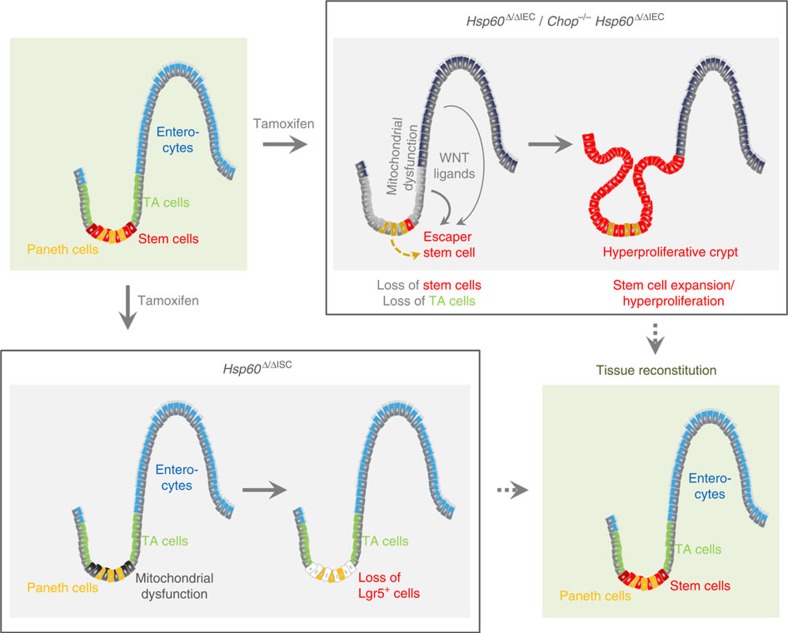
Schematic model of hyperproliferative crypt formation in *Hsp60*^Δ/ΔIEC^ mice. Intestinal crypts loose stem cells and the proliferative capacity in response to HSP60 loss. WNT-related factors are produced by HSP60-deficient IEC including Paneth cells to compensate diminished proliferation. This epithelial microenvironment causes HSP60-positive escaper stem cells to hyperproliferate and form HSP60-positive crypt nodules, finally leading to tissue reconstitution. In contrast, HSP60 deficiency in Lgr5-positive stem cells causes transient loss of stem cells without alterations in tissue morphology.

In conclusion, we demonstrate that HSP60 deficiency induces mitochondrial dysfunction and MT-UPR activation in the intestinal epithelium, independently of the transcription factor CHOP. Mitochondrial dysfunction is associated with a loss of proliferative capacity and stemness in IEC, concomitant with a compensatory release of WNT-related signals. This microenvironment leads to a hyperproliferative response of residual stem cells that escaped *Hsp60* deletion, leading to tissue reconstitution and demonstrating mitochondrial function to control stem cell homeostasis (Fig. 10).

## Discussion

In previous studies with IBD patients, we and others demonstrated the presence and clinical relevance of activated ER UPR pathways in IEC^16^,^17^,^18^. Moreover, we demonstrated the organelle-specific induction of chaperones including GRP78 and HSP60 in the epithelium of IBD patients and mouse models of colitis^30^. The aim of the present study was to investigate the role of the mitochondrial chaperone HSP60 in the regulation of epithelial cell homeostasis by the use of conditional knockout mice.

Epithelial-specific HSP60 deficiency-induced MT-UPR activation in the absence of inflammation-related tissue pathology. Most importantly, HSP60 deficiency caused mitochondrial dysfunction accompanied by impaired proliferation of the intestinal epithelium and loss of stemness. We hypothesize that an inadequate cellular energy supply due to mitochondrial dysfunction and impaired ATP production might antagonize proliferation of stem and progenitor cells in the crypt compartment. Anti-proliferative effects of HSP60 deficiency have been shown in the kidney cell line HEK293 (ref. 42), however, the underlying mechanisms and relevance have not yet been resolved. We expected the UPR-related transcription factor CHOP to be involved, since CHOP has been shown to induce cell cycle arrest^43^. Recently, we generated epithelial-specific *Chop* transgenic mice and demonstrated that high levels of CHOP attenuate cell cycle progression leading to delayed epithelial proliferation and wound closure in response to colonic injuries^34^. Nevertheless, using *Chop*^−/−^ *Hsp60*^Δ/ΔIEC^ mice in this study did not indicate any impact of CHOP-mediated signals on mitochondrial dysfunction-associated loss of proliferation and stemness. Thus, CHOP-mediated target functions are most likely cell-context dependent, including differences in the phosphorylation status of the protein and/or the availability of binding partners.

In this study, HSP60 deficiency reduced the numbers of *Olfm4-* and *Lgr5-*positive stem cells. This is remarkable and might point towards a fundamental role of organelle-specific stress regulation, since the same phenomenon has been reported for the stem-cell-specific deletion of the ER chaperone *Grp78*. However, in the latter study chaperone deficiency caused a loss of stemness involving the PERK-eIF2α-CHOP branch of the ER-UPR. The authors concluded that UPR signalling plays an important role in the regulation of intestinal epithelial stem cell differentiation^19^. In another study, hypomorphic function of the ER-UPR transcription factor Xbox binding protein 1 (XBP-1) led to an expansion of the *Olfm4*- and *Lrg5*-positive stem cell zone, suggesting that the IRE1α-XBP-1 branch of the ER-UPR contributes to proliferative control^44^. In the context of mitochondrial chaperone deficiency, organelle dysfunction and loss of stemness seem independent of CHOP-mediated signals. In line, the MT-UPR mediated regulation of hematopoietic stem cell aging was dependent on the interplay of the histone deacetylase sirtuin 7 (SIRT7) and the transcription factor Nuclear Respiratory Factor 1 (NRF1)^21^.

HSP60 deficiency in the tamoxifen induced *Villin*CreER^T2-Tg^ driven model is not complete due to sporadic failure of Cre-mediated *Hsp60* deletion. The appearance of escaper cells in the crypt bottom is a feature of *Villin*CreER^T2-Tg^ mice and has been reported before^45^. HSP60-positive crypt foci are characterized by an accumulation of Ki67- and OLFM4-positive cells, suggesting that escaper stem cells in the crypt compartment hyperproliferate in response to changes in the epithelial microenvironment deficient in HSP60. In fact, stem and progenitor cell homeostasis is a tightly regulated process involving growth promoting factors released by Paneth cells as well as the surrounding mesenchyme^46^. Paneth cell factors that have been described to constitute a niche for stem cell proliferation are WNT3, WNT6, WNT9B and RSPO1 (refs 47, 48, 49). These mediators are complemented by mesenchymal factors like WNT2B, WNT4 and WNT5A (refs 47, 50). Yet, the significance of epithelial versus mesenchymal contributions is still a matter of debate^40^,^51^,^52^,^53^ and most probably, a tight interplay between growth-regulating factors originating from different cell types of the intestine occurs. In our setup, we cannot exclude the possibility that IEC are not the sole source of enhanced production of WNT-associated factors, in particular RSPO1. Mesenchymal cells such as fibroblasts might contribute to the hyperproliferative microenvironment induced by the loss of HSP60, and subsequent mitochondrial dysfunction, in IEC. However, our data indicate profound changes in the expression of WNT-related factors including RSPO1 in functionally impaired IEC and changes in the surrounding tissue are most likely a consequence of IEC-derived signals.

Disturbed cryptal homeostasis and the appearance of stem cell hyperproliferation have been reported in literature for two distinct circumstances. First, aberrant growth signalling in the stem cells themselves leads to uncontrolled proliferation as seen in the APC^min^ (ref. 54) or the *Rspo1* transgenic mouse model^55^. Second, as demonstrated in irradiation-mediated epithelial eradication, stem cell hyperproliferation occurs in response to epithelial damage, aiming to reconstitute tissue morphology. Few surviving stem cells build up new crypts by proliferative expansion before differentiation limits stem cells back to the crypt bottom^56^. The knockout of essential epithelial genes can be considered as a comparable condition, since the escaper cells are surrounded by compromised cells, which provide the escapers with a proliferative advantage. Examples for this phenomenon are the epithelial-specific deletion of the stem cell marker *Ascl-2* (ref. 57), the transcriptional activator *c-Myc* (ref. 58) and the β-Catenin binding partner TCF4 (ref. 59). In all cases, the proliferative response of escaper cells aims to restore homeostasis. Using the *Hsp60*^Δ/ΔIEC^ mouse model, we suggest a paracrine mechanism by which the compromised crypt epithelium sets up a microenvironment that triggers hyperproliferation of escaper stem cells. We identified WNT10A as a new relevant factor in the control of the proliferative capacity of IEC that is highly induced in HSP60-deficient Paneth cells and was able to promote growth of intestinal organoids suffering from mitochondrial dysfunction.

In conclusion, mitochondrial dysfunction induced by HSP60 loss antagonized intestinal stem cell homeostasis through CHOP-independent signalling mechanisms. HSP60 deficiency in IEC triggered the paracrine release of WNT-related signals associated with hyperproliferation of residual stem cells that escaped *Hsp60* deletion, demonstrating a fundamental role of mitochondrial function in the control of intestinal stem cell homeostasis. In cases where this homeostasis is constantly challenged, such as under conditions of chronic inflammation, this mechanism might contribute to intestinal tumorigenesis.

## Methods

### Ethics statement

The maintenance and breeding of mouse lines and all experiments were approved by the Committee on Animal Health and Care of the local government body of the state of Upper Bavaria (Regierung von Oberbayern; TVA 12-12 and TVA 214-13) and performed in strict compliance with the EEC recommendations for the care and use of Lab. Anim. (European Communities Council Directive of 24 November 1986 (86/609/EEC)).

### Animals

Conditional *Hsp60* knockout mice were generated by Taconic-Artemis (Cologne, Germany) in close consultation with our lab as follows: Mouse genomic fragments of the *Hsp60* locus were subcloned using RPCIB-731 BAC library via ET recombination and recloned into a basic targeting vector placing a F3-site flanked Puromycin resistance cassette in intron 3 and a thymidine kinase cassette downstream of the 3′ UTR. LoxP sites flanked exons 4 to 8 (chaperone ATPase domain). The targeting vector was sequenced to confirm correctness. The linearized DNA vector was electroporated into C57BL/6N embryonic stem cells, Puromycin selection (1 μg ml^−1^) started on day 2 and counterselection with Gancyclovir (2 μM) started on day 5 after electroporation. Embryonic stem cell clones were isolated on day 8 and analysed by Southern blotting according to standard procedures. Blastocysts were isolated from the uterus of Balb/c females at day 3.5 *post coitum* and 10–15 targeted C57BL/6NTac embryonic stem cells were injected into each blastocyst. After recovery, 8 injected blastocysts were transferred to each uterine horn of 2.5 days *post coitum*, pseudopregnant NMRI females. Chimerism of offspring was measured by coat colour contribution of embryonic stem cells to the Balb/c host (black/white). Highly chimeric mice were bred to strain C57BL/6 females transgenic for the Flp recombinase gene (Flp-Deleter) to remove the Puromycin resistance cassette in mice carrying the conditional knockout allele (*Hsp60*^flox/WT^). Germline transmission was identified by the presence of black strain C57BL/6 offspring.

*Hsp60*^flox/flox^ mice were crossed with various Cre transgenic mice to generate cell-type-specific *Hsp60* knockout mice. *Villin*Cre^Tg^ and *Villin*CreER^T2-Tg^ mice (both C57Bl/6N) were provided by Klaus Peter Janssen^35^. *Lgr5*CreER^T2^-IRES-*Egfp*^Tg^ mice (C57Bl/6J^60^) and *Chop*^−/−^ mice (C57Bl/6J) mice were both purchased from Jackson lab (Bar Harbor, ME). All mice were bred over several generations in our animal facility to harmonize the intestinal microbiota.

### Induction of postnatal recombination and monitoring of mice

Phytoestrogen free pellets (ssniff, Soest, Germany) were fed for 2 weeks to male 8 weeks old *Hsp60*^flox/flox^ X *Villin*CreER^T2-Tg^ or *Hsp60*^flox/flox^ X *Lgr5*CreER^T2^ *Egfp*^Tg^ and their appropriate control mice, respectively. Afterwards, they received 400 mg tamoxifen citrate (Tam) per kg chow feed (LASvendi, Soest, Germany) in pellets *ad libitum* for 7 days. Body weight was monitored before, during and after oral administration of tamoxifen. Body weight, general condition, behaviour and intestinal symptoms were assessed by a score between 0 and 10 each according to the approved application for animal experiments. The animals were killed by CO_2_ inhalation at the indicated time points or after reaching an affection index of 20, respectively.

### Embryo preparation and genotyping

*Hsp60*^flox/WT^ X *Villin*Cre^Tg^ mice were mated with *Hsp60*^flox/flox^ mice. When females were plug positive, embryos were assumed to be in developmental stage E 0.5 (0.5 days *post coitum*). At the indicated stage (E12.5–13.5) dams were killed by cervical dislocation and the uterus was removed. The embryos and the surrounding amniotic sac (visceral endoderm) were prepared from the uterus, examined under the microscope, killed and tails were taken for genotyping.

For genotyping, tail cuts or ear punches were lysed in a 10 mM Tris-HCl buffer pH 8.0 buffer containing 50 mM KCl, 0.45% Nonidet P40, 0.45% Tween 20 and 0.5 mg ml^−1^ Proteinase K overnight (O.N.) at 65 °C and inactivated at 95 °C for 10 min. Two microlitre of the clear supernatant was used for Crimson-Taq PCR (NEB, Ipswich, MA). For genotyping of jejunal villi and crypts of *Hsp60*^Δ/ΔIEC^ mice 10 μm thick cryosections were H&E stained (Harris formulation), 50,000 μm^2^ cells were cut using the laser-dissection microscope (Leica, Soest, Germany) and lysed in RLT Plus buffer (Qiagen, Hilden, Germany). DNA was isolated using the Allprep DNA/RNA Mini kit (Qiagen). Two microlitre of DNA were used for Crimson-Taq PCR. Primers used for genotyping are given in Supplementary Table 1. All uncropped agarose gels can be found in Supplementary Fig. 11.

### Tissue processing and staining procedures

The intestine was removed immediately after killing, trimmed free of adjacent tissue and cleaned of stool. Parts of the gastrointestinal tract were cut open and prepared as a ‘swiss role'^61^, fixed in 4% PBS buffered formaldehyde, dehydrated and embedded in paraffin. 5 μm sections were stained with hematoxylin (of Mayer) and 0.2% eosin (ethanolic solution; both Medite, Burgdorf, Germany) in an automated staining machine (Leica, Soest, Germany).

Immunohistochemical (IHC) and immunofluorescence (IF) staining was performed on 5 μm sections of formalin-fixed and paraffin-embedded (FFPE) tissue. Antigen unmasking was performed by cooking the sections in 10 mM Citrate buffer pH 6.0 in a pressure cooker for 23 min. In case of IHC staining, endogenous peroxidases were blocked by 10 min incubation with 3% H_2_O_2_ (Sigma-Aldrich, St Louis, MO). Specimens were blocked in blocking buffer containing 5% serum gained from the host species of the respective secondary antibody. Antibodies and dilutions are given in Supplementary Table 2. For immunizing peptide blocking experiments, anti-RSPO1 was incubated with the appropriate blocking peptide (Sigma-Aldrich) in a ratio of 1:2 for 30 min at room temperature prior application on specimens. For IF staining of OLFM4, the TSA Fluorescence kit (Perkin Elmer) was used to amplify the fluorescence signal according to the manufacturer's instructions. In particular, tissue specimen were incubated with 3% hydrogen peroxide for 10 min after antigen retrieval followed by blocking using a Avidin/Biotin kit (Vector laboratories). Subsequently, after applying the secondary antibody, a biotinylated antibody was added and tissue specimen were incubated for 30 min with an ABC mix (Vectastatin ABC kit, Vector laboratories). Finally, Cyanine 5 Tyramide was applied for 5 min before sections were mounted with water based mounting medium.

In the case of IHC, antigen detection was performed using the DAB enhanced liquid substrate system (Sigma-Aldrich). Slides were finally counterstained with hematoxylin. Sections were viewed on a Zeiss Axioskop 40 (Zeiss, Jena, Germany) microscope and imaged using a Zeiss Axiocam and the Axiovision software. For IF, nuclei were stained with DAPI (Sigma-Aldrich) and stainings were visualized using the Flouview FV10i microscope (Olympus, Shinjuku, Japan). For detection of apoptosis in FFPE tissue sections the Apo-BrdU *In Situ* Fragmentation Assay Kit (BioVision, Milpitas, CA) was used according to the manufacturer's instructions. For positive controls, 5 μm thick tissue sections were treated with DNaseI (Macherey-Nagel, Düren, Germany) for 15 min immediately after antigen retrieval.

For *in situ* hybridization, Digoxigenin (DIG)-labelled antisense and appropriate sense control RNA probes were generated from cDNA-containing vectors by *in vitro* transcription using T3, T7, or SP6 RNA polymerase (Promega, Mannheim, Germany). The following probes were used: Olfm4 (GenBank Accession number NM_001030294.1, 643–1458; kindly provided by Hans Clevers, Utrecht) and Axin2 (NM_015732.4, nucleotides 3472–4256). *In situ* hybridization was performed essentially as described previously^60^. Briefly, paraffin-embedded tissue sections were treated with proteinase K, post-fixed and hybridized overnight at 70 °C. After washing and blocking, sections were incubated overnight at 4 °C with preabsorbed alkaline phosphatase-conjugated anti-digoxigenin (1:2,000, Roche). Colour reaction was performed with BM Purple AP substrate (Roche).

### Electron microscopy

One centimetre long segments of the jejunum or distal colon from *Hsp60*^Δ/ΔIEC^ mice and according controls were fixed in 2.5% glutaraldehyde (Electron Microscopy Sciences, Hatfield, PA). After washing in buffered saccharose and osmication for 1 h, tissues were dehydrated in acetone and then processed for Epon embedding. The tissues were then trimmed so that ultrathin sections of gut rings could be cut and counterstained with uranyl acetate and lead citrate. Electron micrographs from each gut specimen were obtained at 19,000-fold magnification from each animal. Of these, up to nine images for each specimen and animal were randomly selected and mitochondrial abundance and diameter were measured using the analySIS Dock System (Soft Imaging System GmbH, Münster, Germany).

### Isolation of total IEC and villus tip/crypt bottom IEC

Primary IEC were purified as previously described^62^. Approximately 7 cm of intestine were inverted on a needle, vortexed vigorously and incubated (37 °C, 15 min) in DMEM containing 10% fetal calf serum (FCS superior, Biochrom, Berlin), 1.0% Glutamine, 0.8% antibiotics/antimycotics (all Sigma-Aldrich, St Louis, MO) supplemented with 1 mM dithiothreitol (Roth, Karlsruhe, Germany). The IEC suspensions were centrifuged (7 min, 300*g*, RT) and cell pellets were re-suspended in DMEM containing fetal calf serum, L-glutamine and antibiotics. The remaining tissue was incubated in 20 ml PBS (10 min, 37 °C) containing 1.5 mM EDTA (Roth). Thereafter, the tissue was discarded and the cell suspension from this step was centrifuged as mentioned above. Finally, primary IEC were purified by centrifugation through a 20%/40% discontinuous Percoll gradient (GE Healthcare, Uppsala, Sweden) at 600*g* for 30 min.

The isolation of primary jejunal villus tip IEC was adapted from a protocol published by Ferraris *et al*.^63^ and Mariadason *et al*.^64^. The jejunum was cut in two ∼5 cm pieces, inverted, cleaned of stool and beaded on a needle. Gut tissue was incubated (15 min, 37 °C) in citrate buffer (96 mM NaCl, 1.5 mM KCl, 27 mM NaCitrate, 8 mM KH_2_PO_4_, 5.6 mM Na_2_HPO_4_, pH 7.3) to vigorously remove mucus and stool remnants. The gut tissues fixed on the needles were then transferred into 10 ml of isolation buffer (1.5 mM EDTA, 0.5 mM DTT, 1 mg ml^−1^ BSA) and incubated for 10 min at 37 °C while rocking (fraction 1). Gut tissues were transferred into 10 ml fresh isolation buffer and incubated as above (fraction 2). Fractions 1 and 2 of each jejunum were centrifuged (300*g*, 5 min, 4 °C), re-suspended and combined in 1 ml cold PBS and re-centrifuged (300*g*, 5 min, 4 °C). To gain fractions 3–10, the isolation procedure was repeated for 6, 5, 5, 9, 10, 25 and 30 min always transferring the gut to a fresh tube with isolation buffer. Fraction 10 refers to the crypt bottom IEC. IEC pellets of isolated primary IEC were lysed in 350 μl RA1 buffer (Macherey-Nagel, Düren, Germany) for subsequent RNA and/or protein isolation. Cell purity was assessed by determining the absence of CD3-positive T-cell contaminations and the presence of E-Cadherin as an epithelial cell marker, respectively.

### LC–MS/MS amino-acid analysis of plasma samples

EDTA blood was taken during sampling from venacava. Quantitation of amino acids in EDTA plasma was performed using LC–MS/MS with aTRAQ labelling (aTRAQ reagent kit 4442671, AB SCIEX, Framingham, MA, USA). Quantitation was done according to the manufacturer's instructions using 40 μl of serum sample. The analysis was performed on a triple quadrupole QTRAP3200 LC–MS/MS system (AB SCIEX) coupled to an Agilent 1260 Infinity Quaternary LC Pump (Agilent, Santa Clara, CA, USA).

### Quantitative real-time PCR and western blotting

Reverse transcription was performed using 1 μg total RNA and a MMLV reverse transcriptase with point mutation (Promega, Mannheim, Germany). Quantitative Real-time PCR (qRT–PCR) was performed using 1 μl cDNA in a Light Cycler 480 system (Roche Diagnostics, Mannheim, Germany) applying the Universal Probe Library system according to the manufacturer's instructions. Primer sequences were according to Probe Finder software (Roche, Mannheim, Germany). Relative induction of gene mRNA expression was calculated using the Light Cycler 480 software using the expression of *Gapdh* for normalization. Data were expressed as fold change compared with *Hsp60*^flox/flox^ mice. Primers used for qRT–PCR are given in Supplementary Table 3.

For western blot analysis, purified protein pellets isolated from primary IEC or organoids (Macherey-Nagel, Düren, Germany) were suspended in lysis buffer containing 7 mol l^−1^ urea, 2 mol l^−1^ thiourea, 2% CHAPS, 1% DTT (all from Roth, Karlsruhe, Germany) and protease inhibitor (Roche Diagnostics, Mannheim, Germany) and homogenized by ultrasonication. Total protein concentrations were determined using BioRad protein assay (Munich, Germany). Samples were diluted with 5 × SDS buffer and 25 μg of protein were subjected to electrophoresis on 10% SDS–PAGE gels. Proteins were transferred to PVDF membranes (Millipore, Billerica, MA) using a semi dry blotting chamber (Peqlab, Erlangen, Germany). Thereafter, membranes were incubated in TBST containing 5% skim milk powder for blocking. Antibodies and dilutions are given in Supplementary Table 2. Antibodies were applied for O.N. incubation at 4 °C. Appropriate HRP-conjugated secondary antibodies goat anti-rabbit and rabbit anti-goat (both from Dianova, Hamburg, Germany) were used to detect the respective immunoreactive protein using an enhanced chemiluminescence light-detecting kit (GE, Arlington Heights, IL).

### Intestinal organoid culture

Small intestinal crypt organoids were isolated by incubation of mucosal scratches in 2 mM EDTA buffer for 30 min at 4 °C. Serial shaking steps resulted in fractions containing villi, crypts and mesenchymal cells. Fractions enriched in crypts were identified via light microscopy and filtered through a 70 μm nylon mesh. Crypt fractions were centrifuged (300*g*, 5 min) and embedded in Matrigel BD Biosciences, Franklin Lakes, NJ) for cultivation as previously described^40^,^48^. Organoids from *Hsp60*^flox/flox^ X *Villin*CreER^T2-Tg^ and *Hsp60*^flox/flox^ control mice were cultivated in an advanced DMEM/F12 medium (Gibbco, Cincinnati, OH) containing 2 mM GlataMax (Gibbco), 10 mM HEPES, penicillin, streptomycin and amphotericin (all Sigma-Aldrich, St Lois, MO) supplemented with N2, B27 (both Gibbco), 1 mM *N*-acetylcystein (Sigma-Aldrich), 50 ng ml^−1^ EGF (ImmunoTools, Friesoythe, Germany), 100 ng ml^−1^ noggin and 1 ng ml^−1^ R-spondin 1 (both PeproTec, Rocky Hill, NJ). Organoids were passaged every 6–7 days and embedded in fresh Matrigel. *Ex vivo* induction of the *Hsp60* knockout was achieved by adding 1.5 μl of 100 μM (Z)-4-hydroxytamoxifen (4-OHT; LKT, St Paul, MN) to 300 μl culture medium per well of a 48 well plate. When indicated, recombinant murine WNT10A (Cloud-Clone Corp., Houston, TX) or EUK-134, a synthetic superoxide dismutase (SOD)/catalase mimetic (Sigma-Aldrich), were added in a concentration of 100 ng ml^−1^ (WNT10A) and 100 μM (Euk-134), respectively. Growth measurements were performed using a Olympus CK X 41 microscope and Olympus cellSens Entry software.

### Measurement of living cells and cellular ATP content

ATP content of organoids was measured using the CellTiter-Glo Luminescent Cell Viability Assay (Promega, Mannheim, Germany). The activity of life- and dead-cell proteases was measured using the MultiTox-Fluor Cytotoxicity Assay (Promega, Mannheim, Germany) according to the manufacturer's instructions in a 96-well format. Life and death cell protease activity as well as ATP were measured in the same wells.

### Measurement of mitochondrial respiration

Oxygen consumption of organoids was measured with high-resolution respirometry (Oxygraph-2k, Oroboros Instruments, Austria). Organoids were pipetted into 2 ml MIR05 buffer (110 mM sucrose, 60 mM potassium lactobionate, 0.5 mM EGTA, 3 mM MgCl_2_*6H_2_O, 20 mM taurine, 10 mM KH_2_PO_4_, 20 mM HEPES, 1 g l^−1^ BSA-fatty acid free, pH 7.1 at 37 °C, as described by Oroboros Instruments. The stirrer speed was set to 750 r.p.m. Digitonin (2 μM) was added to permeabilize plasma membranes. State 4 respiration was measured in the presence of succinate/rotenone (5 mM/2 μM). By addition of ADP (5 mM) phosphorylating state 3 was induced. ATP synthase was inhibited and proton leak respiration (state 4o) was determined by addition of oligomycin (2 μg ml^−1^). Non-mitochondrial oxygen consumption determined in presence of the complex III inhibitor antimycin A (2.5 μM) and subtracted from the other respiratory states. Respiratory Control Ratio (RCR) was calculated dividing state 3 by state 4o respiration. All in-plate measurements with organoids were performed in 4–6 wells per treatment. Data represent one of three independent experiments with *N*=3 individual mice per genotype.

### Statistics

Data of 3–6 animals per experimental group are indicated. Statistically significant differences were determined by the parametric unpaired *t*-test or by one-way analysis of variance followed by appropriate *post hoc* tests, respectively (treatment versus control group(s)). If the data did not fulfil the prerequisites of parametric statistics (not normally distributed), a Mann–Whitney rank sum test or a Kruskal–Wallis test on ranks followed by Dunn's test, respectively, was performed. To determine differences in the distribution of *de novo* crypt formation among experimental groups, a Kruskal–Wallis test on ranks followed by Dunnś test was performed. Differences reached statistical significance with *P* values<0.05 (*), <0.01 (**) and *P*<0.001 (***). Correlation analysis was performed according to Pearson test. A negative correlation coefficient (*r*) indicates inverse correlations. Statistical computations were performed using Prism software (Graph Pad, La Jolla, CA) and SigmaStat software (Systat).

### Data availability

The authors declare that all data supporting the findings of this study are available within the article and its Supplementary Information Files or from the corresponding author on reasonable request.

## Additional information

**How to cite this article:** Berger, E. *et al*. Mitochondrial function controls intestinal epithelial stemness and proliferation. *Nat. Commun.* **7,** 13171 doi: 10.1038/ncomms13171 (2016).

## Supplementary Material

Supplementary Information Supplementary Figures 1-13 and Supplementary Tables 1-3
