# Supplementary material for: Mitochondrial function controls intestinal epithelial stemness and proliferation
Source: Nat Commun. 2016 Oct 27;7:13171. doi: 10.1038/ncomms13171 (PMC5080445; doi:10.1038/ncomms13171)
Supplement: Supplementary Information — Supplementary Figures 1-13 and Supplementary Tables 1-3 [file ncomms13171-s1.pdf]

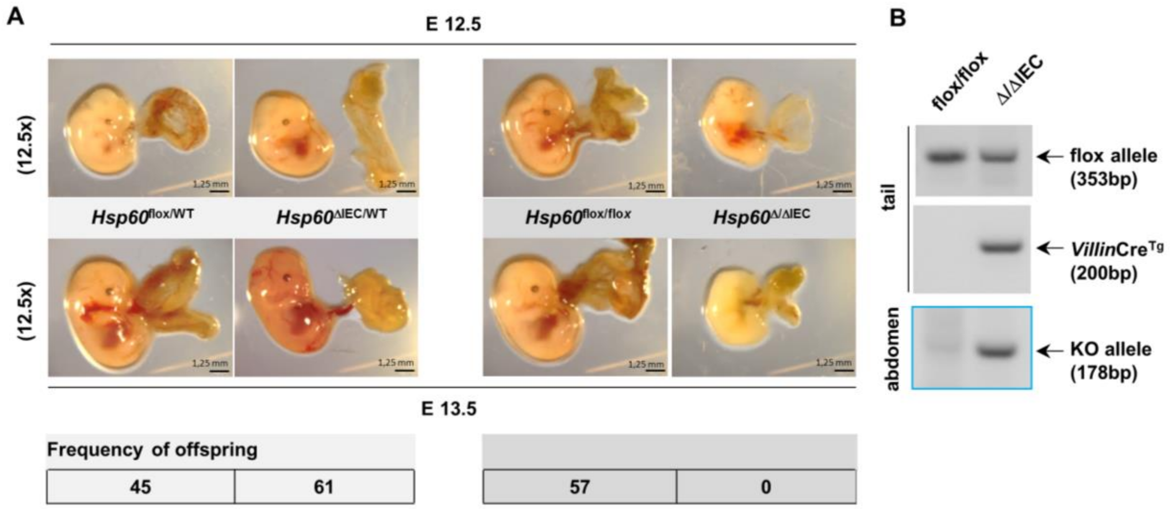

Supplementary Figure 1: *Hsp60*$^{\Delta/\Delta IEC}$ mice are embryonically lethal

(A) Light microscopic pictures show mouse embryos at developmental stage E12.5 and E13.5 prepared from uteri of dams and subsequently genotyped. The visceral yolk sac is placed right next to the embryos. The table shows the frequency of offspring carrying the corresponding genotypes. (B) Representative agarose gels validating the *Hsp60* knockout in embryonic abdominal samples of *Hsp60*$^{flox/flox}$ X *Villin*Cre^{Tg} mice.

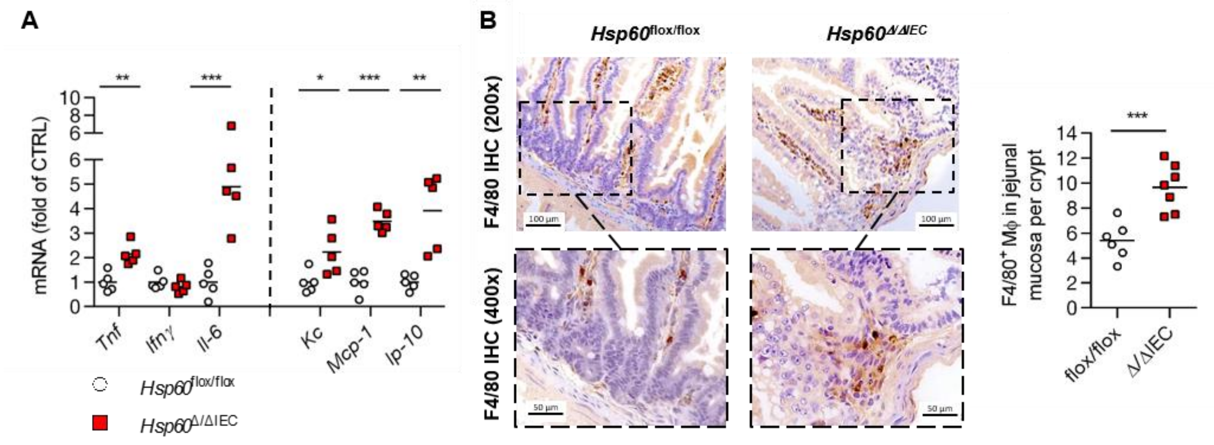

Supplementary Figure 2: Mild signs of mucosal inflammation in jejunum of *Hsp60*$^{\Delta/\Delta IEC}$ mice

(A) qRT-PCR analysis of cytokines and chemokines was performed on mRNA isolated from whole jejunal tissue pieces (N=5 per genotype). Statistical analysis was performed via t-test comparing genotypes. (B) Representative pictures including detailed images show macrophage infiltrates into jejunal mucosa. Quantification of infiltrating F4/80-positive macrophage into jejunal mucosa (evaluation of 20 crypts of one representative mouse per genotype). Lines in the dot plots indicate mean numbers. Asterisk indicate significant differences *P<0.05; **P<0.01; ***P<0.001.

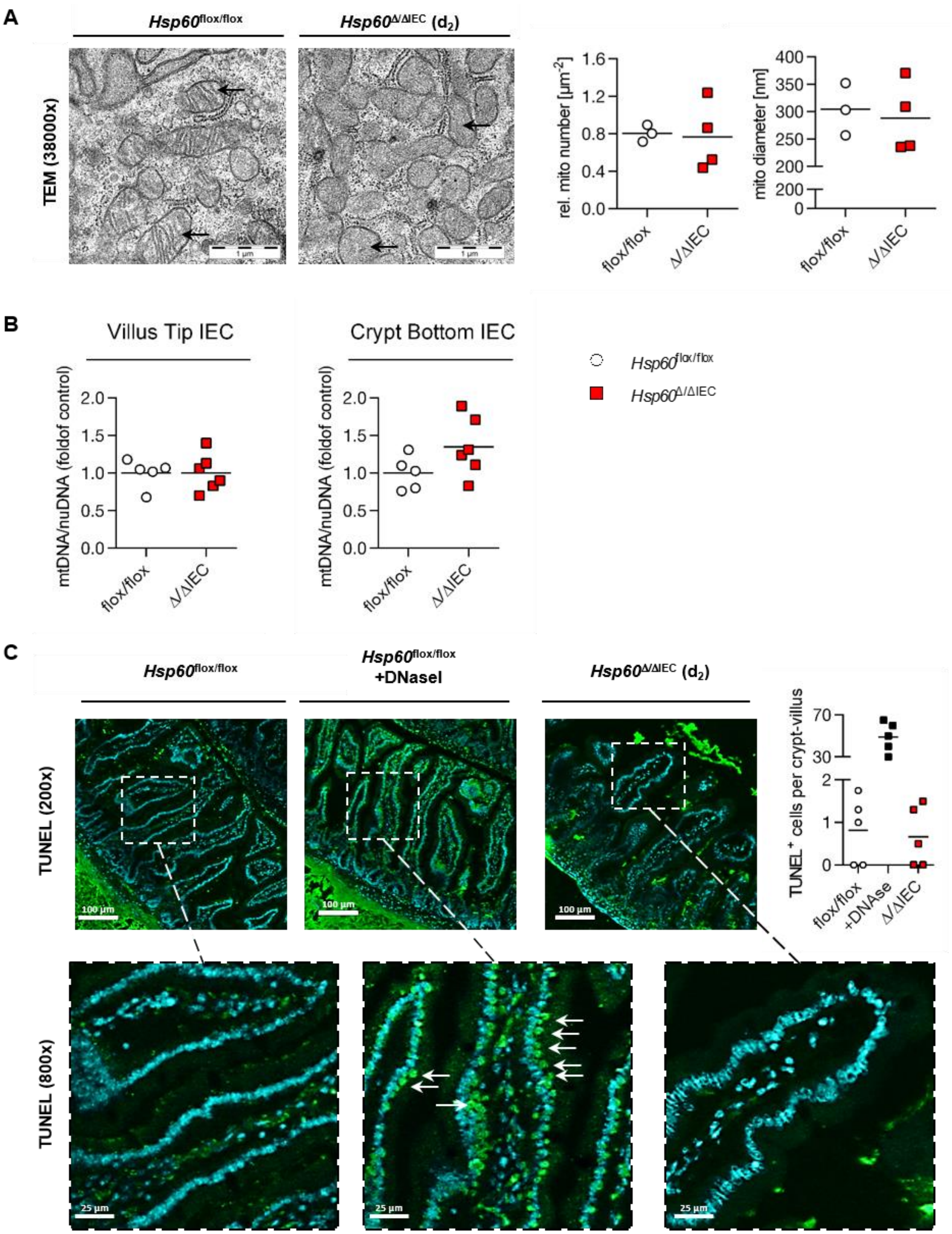

Supplementary Figure 3: HSP60 deficiency does not induce epithelial apoptosis or a loss of mitochondria

(A). The number and diameter of mitochondria in villus and crypt IEC was assessed by electron microscopy of jejunal sections. Arrows indicate differences in mitochondrial christae formation in in *Hsp60*$^{\Delta/\Delta IEC}$ vs. CTRL mice. Right: Mitochondrial numbers and diameter in *Hsp60*$^{\Delta/\Delta IEC}$ mice (N=4) and *Hsp60*$^{flox/flox}$ control mice (N=3) (B) Ratio of mitochondrial to nuclear DNA measured by qPCR in IEC isolated from villi and crypt of *Hsp60*$^{\Delta/\Delta IEC}$ mice and *Hsp60*$^{flox/flox}$ control mice (N=6). (C) Representative images of jejunal sections from *Hsp60*$^{\Delta/\Delta IEC}$ and *Hsp60*$^{flox/flox}$ control mice stained via TUNEL assay to assess apoptotic DNA fragmentation including detailed images in higher magnification (DAPI stains nuclei in cyan). Quantification of TUNEL positive cells per crypt-villus unit (N=5). Positive controls from DNaseI treated tissue sections are indicated by white arrows. Lines in the dot plots indicate mean numbers. Statistical analyses were performed via t-tests comparing genotypes.

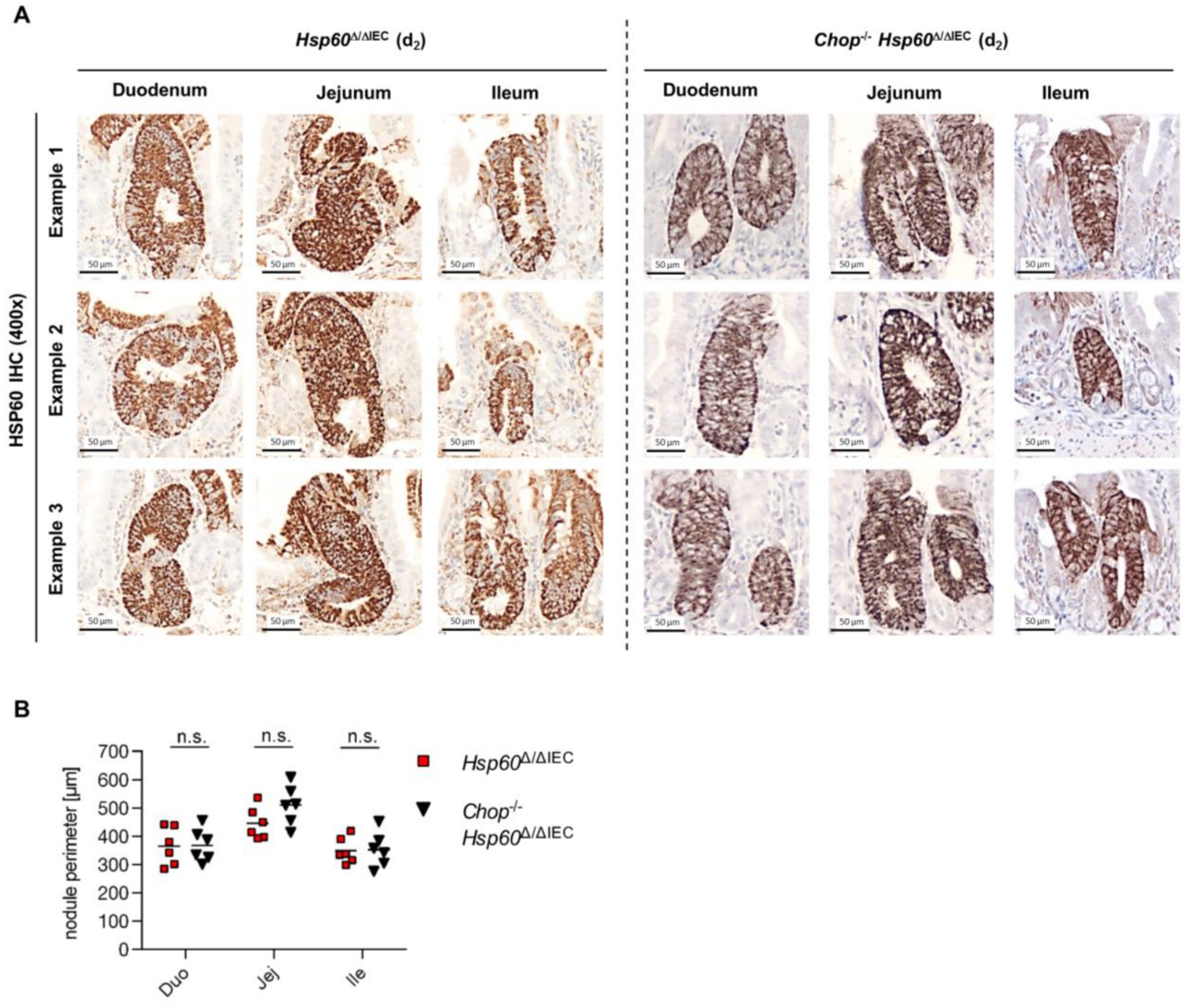

Supplementary Figure 4: Quantification of hyperproliferative, HSP60-positive crypt nodules induced by HSP60 loss in *Hsp60*$^{\Delta/\Delta IEC}$ and in *Chop*$^{-/-}$ *Hsp60*$^{\Delta/\Delta IEC}$ mice

(A) Representative HSP60 IHC stainings of *Hsp60*$^{\Delta/\Delta IEC}$ and *Chop*$^{-/-}$ *Hsp60*$^{\Delta/\Delta IEC}$ mice along the small intestinal tract following proximal to distal compartments. (B) HSP60-positive crypt nodules were quantified along the intestinal tract using HSP60 IHC staining. Comparison of nodule perimeter of HSP60-positive crypt nodules of *Hsp60*$^{\Delta/\Delta IEC}$ and *Chop*$^{-/-}$ *Hsp60*$^{\Delta/\Delta IEC}$ mice (N=6) revealed no differences between genotypes. Lines in the dot plot indicate mean numbers. Statistical analyses were performed via t-tests comparing genotypes.

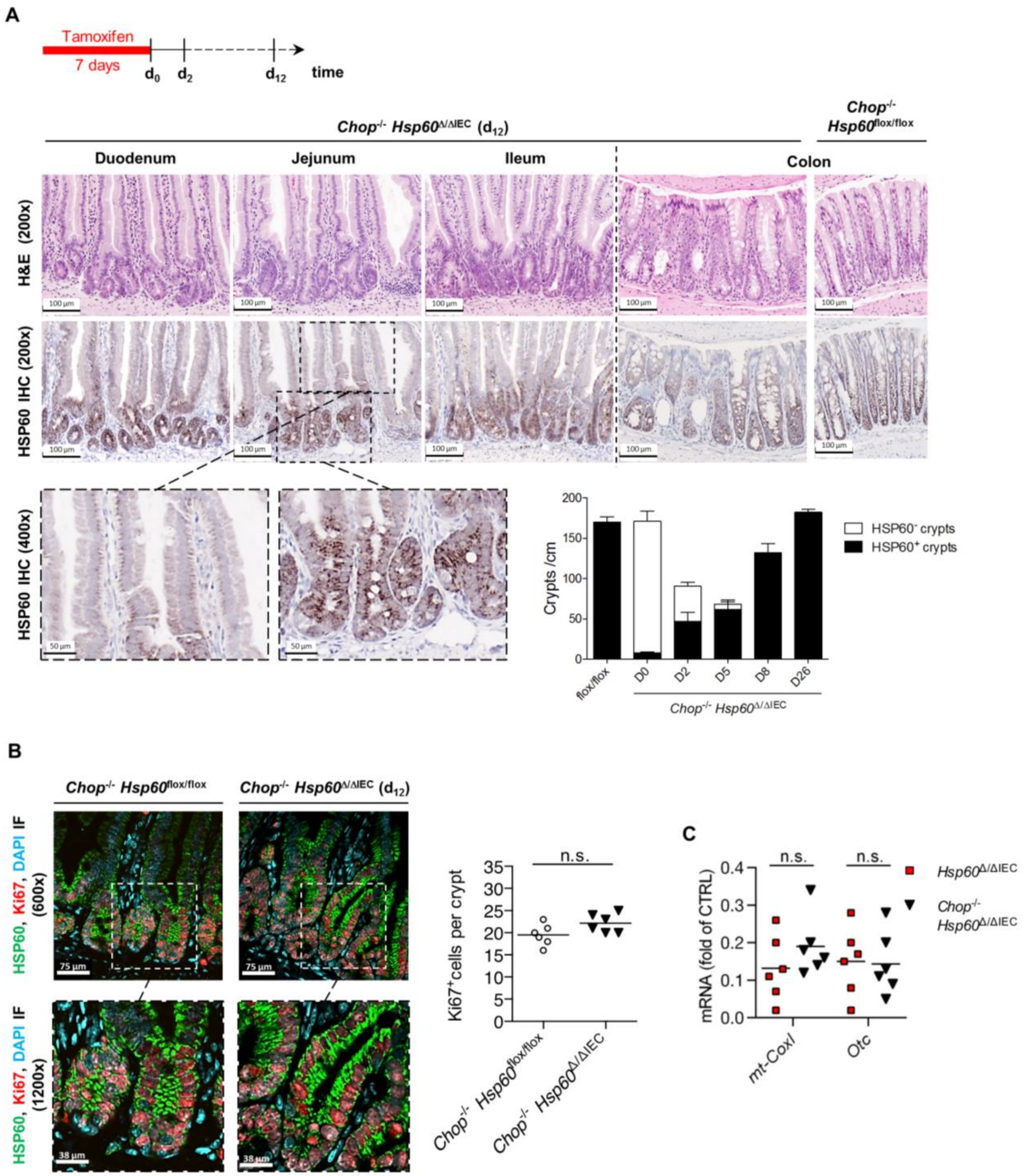

Supplementary Figure 5: Epithelial regeneration in *Chop$^{-/-}$ Hsp60$^{\Delta/\Delta IEC}$* mice

(A) Schedule for oral tamoxifen administration to induce HSP60 deficiency and subsequent regeneration time. Representative H&E and corresponding HSP60 IHC stainings of *Chop*$^{-/-}$ *Hsp60*$^{\Delta/\Delta IEC}$ mice along the intestinal tract following proximal to distal compartments. Detailed images of HSP60 IHC in higher magnification distinguish villus vs. crypt regions of the jejunum. Quantification of HSP60-positive and HSP60-negative crypt numbers of *Chop*$^{-/-}$ *Hsp60*$^{\Delta/\Delta IEC}$ mice (N=4) at different time points after *Hsp60* deletion indicate rapid tissue reconstitution. Bars represent mean +SEM. (B) IF Co-staining of HSP60 (green) and Ki67 (red) on jejunal sections including detailed images in higher magnification of *Chop*$^{-/-}$ *Hsp60*$^{\Delta/\Delta IEC}$ mice and *Chop*$^{-/-}$ *Hsp60*$^{flox/flox}$ controls (DAPI stains nuclei in cyan). Quantification of Ki67-positive cells in HSP60-positive and HSP60-negative crypts (N=5; 2 regions per mouse). (C) qPCR analysis of *mtCoxI* and *Otc* mRNA expression in IEC isolated from the crypt compartment of *Hsp60*$^{\Delta/\Delta IEC}$ and *Chop*$^{-/-}$ *Hsp60*$^{\Delta/\Delta IEC}$ mice. Lines in the dot plots indicate mean numbers. Statistical analyses were performed via unpaired t-tests comparing genotypes.

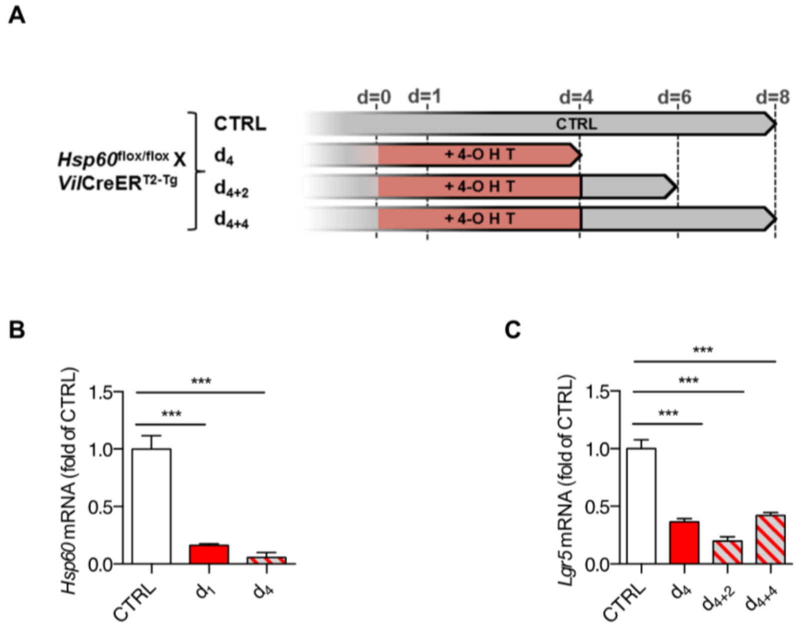

Supplementary Figure 6: Expression of *Hsp60* and *Lgr5* following tamoxifen treatment of organoids

(A) Schematic illustration of the experimental setup using small intestinal organoids. Organoids were isolated from *Hsp60*$^{flox/flox}$, *Villin*CreER$^{T2\text{-}Tg}$ mice and distributed to four protocols. (B) mRNA expression levels of *Hsp60* at d_1 and d_4, respectively, after tamoxifen treatment. (C) qRT-PCR analysis of *Lgr5* mRNA expression after tamoxifen treatment. Bars represent mean +SEM. Asterisk indicate significant differences ***P<0.001. One-Way ANOVA and appropriate post-hoc tests were used for all statistical analyses. Data from organoid experiments derive from at least 3 independent experiments.

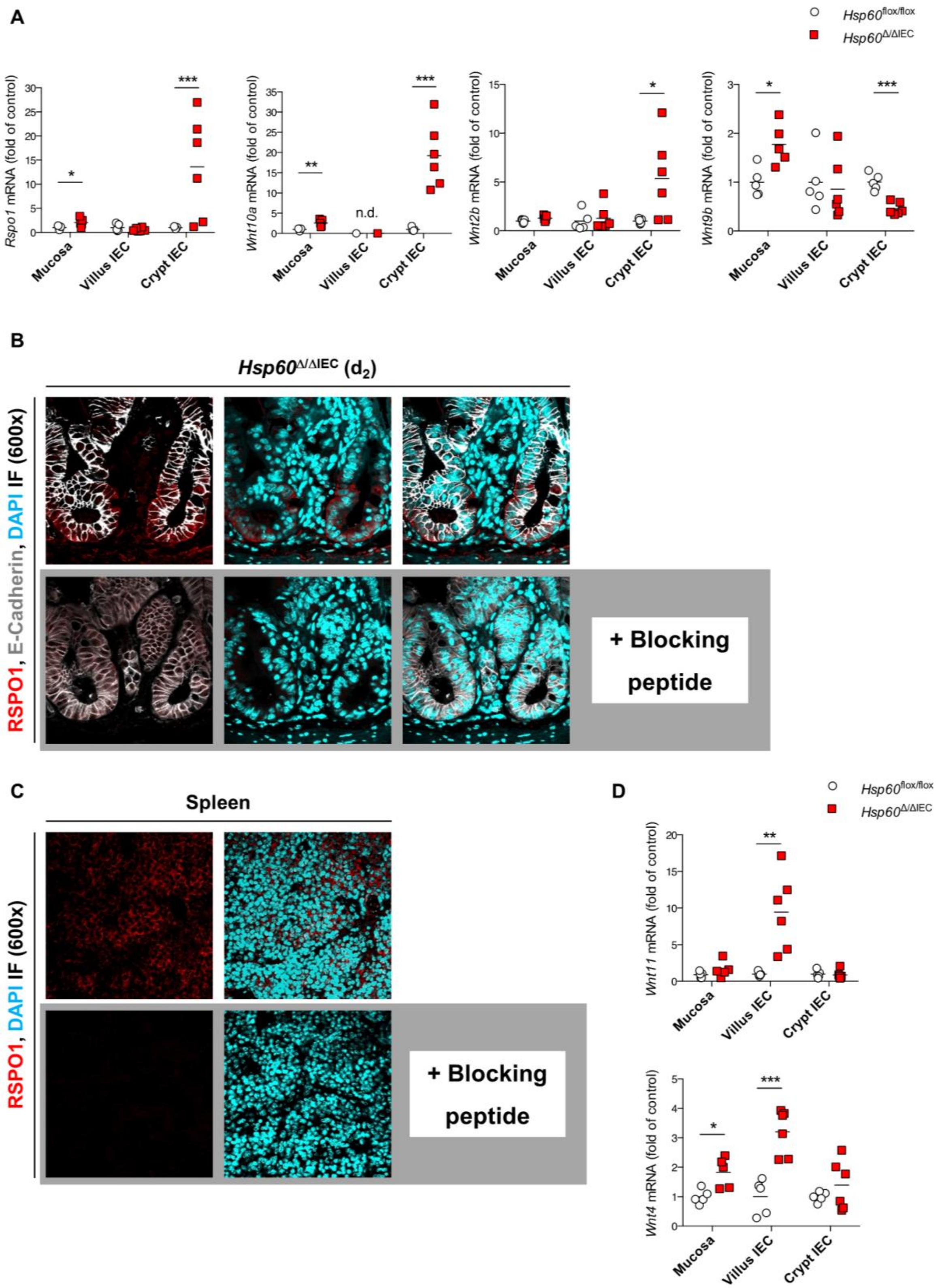

Supplementary Figure 7: Epithelial expression of WNT-related factors and RSPO1 antibody validation

(A) mRNA expression pattern of WNT-related factors found to be significantly altered in crypt-derived IEC from *Hsp60*$^{\Delta/\Delta IEC}$ mice in mucosal tissue (Mucosa), villus-derived IEC and crypt-derived IEC from *Hsp60*$^{flox/flox}$ and *Hsp60*$^{\Delta/\Delta IEC}$ mice. Gene regulation is enriched in crypt-derived IEC fractions. (B) IF stainings of RSPO1 (red) and E-Cadherin (grey) at d_2 in jejunal sections from *Hsp60*$^{\Delta/\Delta IEC}$ mice. A specific blocking peptide was used on consecutive sections (lower panel) with otherwise unchanged staining protocol (DAPI stains nuclei in cyan). (C) IF staining of RSPO1 (red) in splenic sections serving as positive tissue control. A specific blocking peptide was used on consecutive sections (lower panel) with otherwise unchanged staining protocol (DAPI stains nuclei in cyan). (D) mRNA expression pattern of WNT-related factors found to be significantly altered in villus-derived IEC from *Hsp60*$^{\Delta/\Delta IEC}$ mice in mucosal tissue (Mucosa), villus-derived IEC and crypt-derived IEC from *Hsp60*$^{flox/flox}$ and *Hsp60*$^{\Delta/\Delta IEC}$ mice. Gene regulation is enriched in villus-derived IEC fractions. Lines in the dot plots indicate mean numbers. Asterisk indicate significant differences *$P<0.05$; **$P<0.01$; ***$P<0.001$. All statistical analyses were performed via unpaired t-tests comparing genotypes.

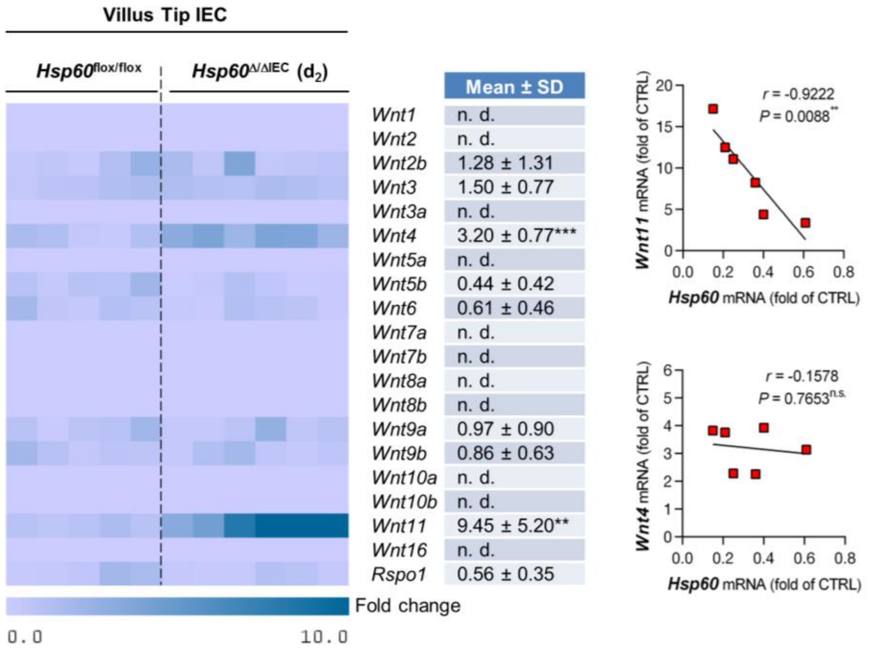

Supplementary Figure 8: HSP60-deficient villus IEC express WNT factors

Left: qRT-PCR analysis of known WNT ligands including the WNT enhancer *Rspo1* in IEC isolated from villus tip epithelium of *Hsp60*$^{\Delta/\Delta IEC}$ (N=6) vs. *Hsp60*$^{flox/flox}$ mice (N=5). Statistical analysis was performed via t-test comparing genotypes at each time point. Asterisk indicate significant differences $^{**}P<0.01$; $^{***}P<0.001$. Right: Correlation analysis (Pearson) of significantly regulated WNT factors compared to HSP60 levels in *Hsp60*$^{\Delta/\Delta IEC}$ mice (the straight line indicates linear regression). P values indicate one-sided significance.

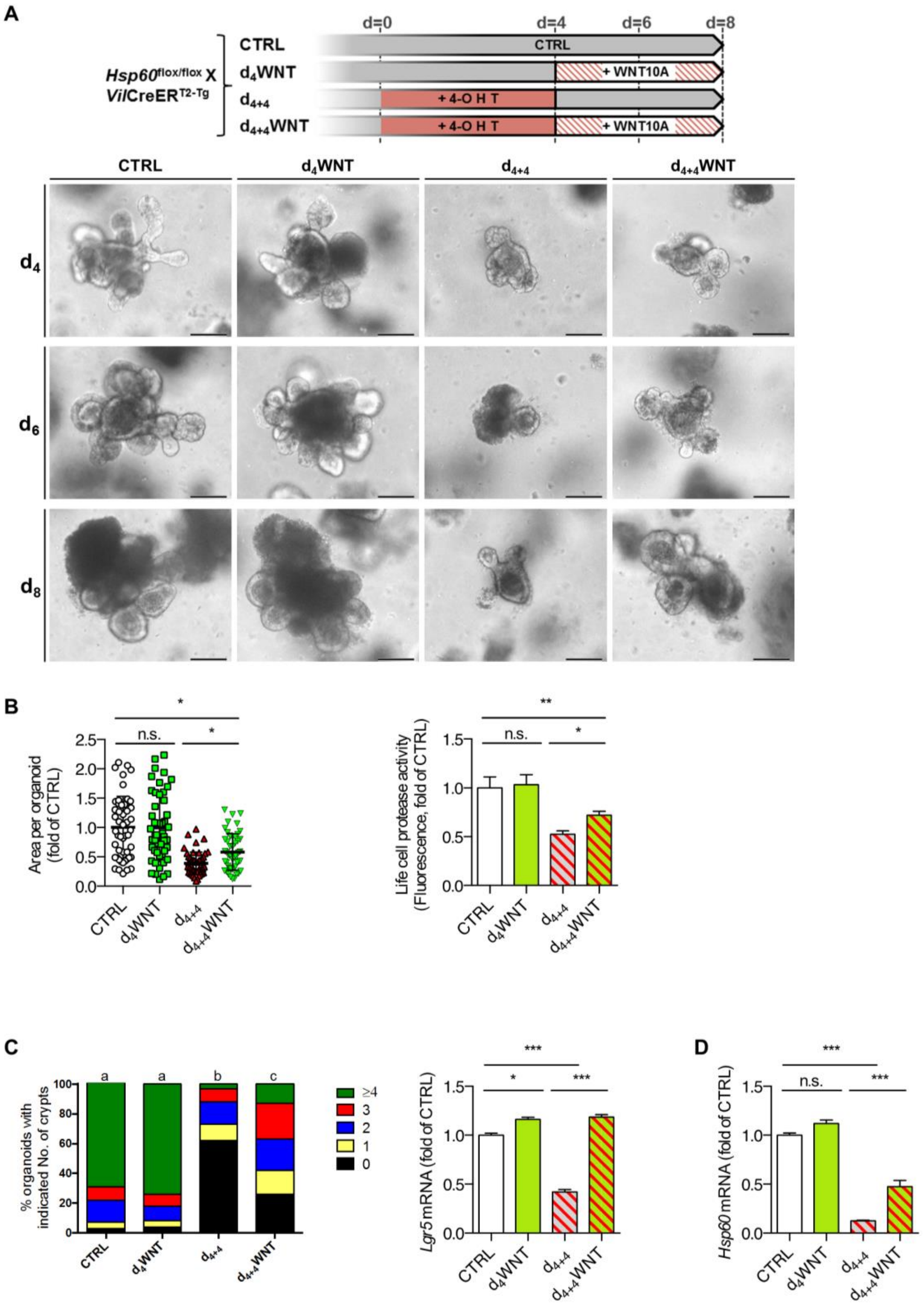

Supplementary Figure 9: WNT10A rescues intestinal organoid growth after HSP60 knockout

Organoids were isolated from *Hsp60*$^{flox/flox}$, *Villin*CreER^{T2-Tg} mice and distributed to four protocols. (A) Experimental scheme to show the effects of d WNT10A supplementation (100ng/mL) on Hsp60 deficient small intestinal organoids. Lower panel: representative pictures of the indicated treatments and time points. (B) Measurement of organoid area (left) and life cell protease activity measured by fluorescence (right) following indicated treatments. (C) Quantification of *de novo* crypt formation (left); a, b, c, significantly different from each other, Kruskal–Wallis test on ranks followed by Dunn´s test. Right: qRT-PCR analysis of *Lgr5* mRNA expression in organoids in response to WNT10A treatment. (D) qPCR analysis of *Hsp60* mRNA expression. Bars represent mean +SEM. Asterisk indicate significant differences *P<0.05; **P<0.01; ***P<0.001. Unless otherwise indicated, One-Way ANOVA and appropriate post-hoc tests were used for all statistical analyses. Data from organoid experiments derive from at least 3 independent experiments.

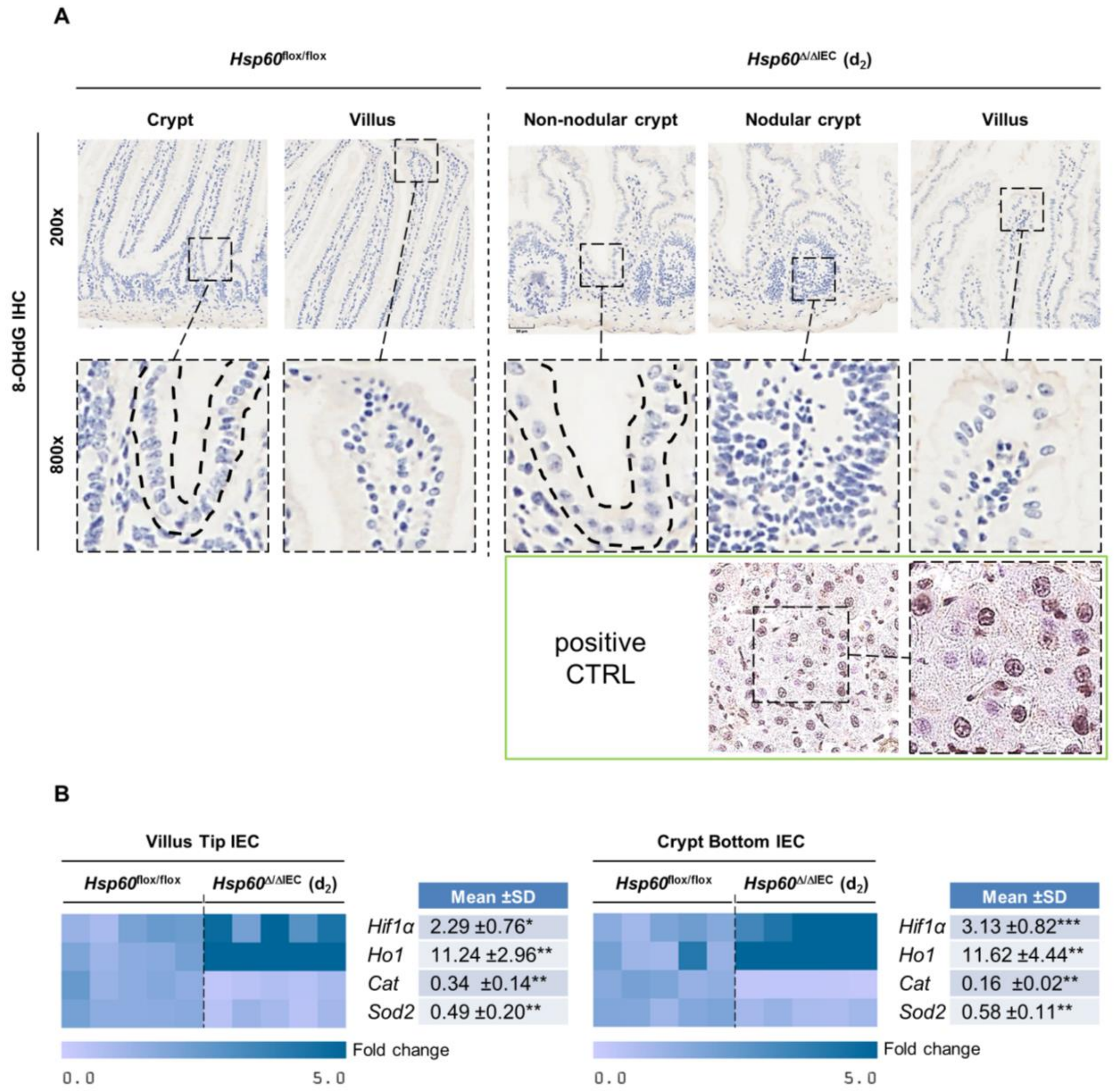

Supplementary Figure 10: Oxidative stress associated with HSP60 deficiency-induced mitochondrial dysfunction seems to play a minor role in IEC

(A) Representative 8-OHdG IHC stainings of *Hsp60*$^{\Delta/\Delta IEC}$ and *Hsp60*$^{flox/flox}$ control mice. Detailed images of 8-OHdG IHC in higher magnification distinguish villus vs. hyperproliferative nodules/ non-nodular crypt regions of the jejunum. Positive control: tissue showing neoplastic changes associated with inflammation. (B) qRT-PCR analysis of oxidative stress-associated genes in IEC isolated from *Hsp60*$^{\Delta/\Delta IEC}$ and *Hsp60*$^{flox/flox}$ control mice (N=5). Asterisk indicate significant differences *P<0.05; **P<0.01; ***P<0.001. Statistical analyses were performed using unpaired t-test.

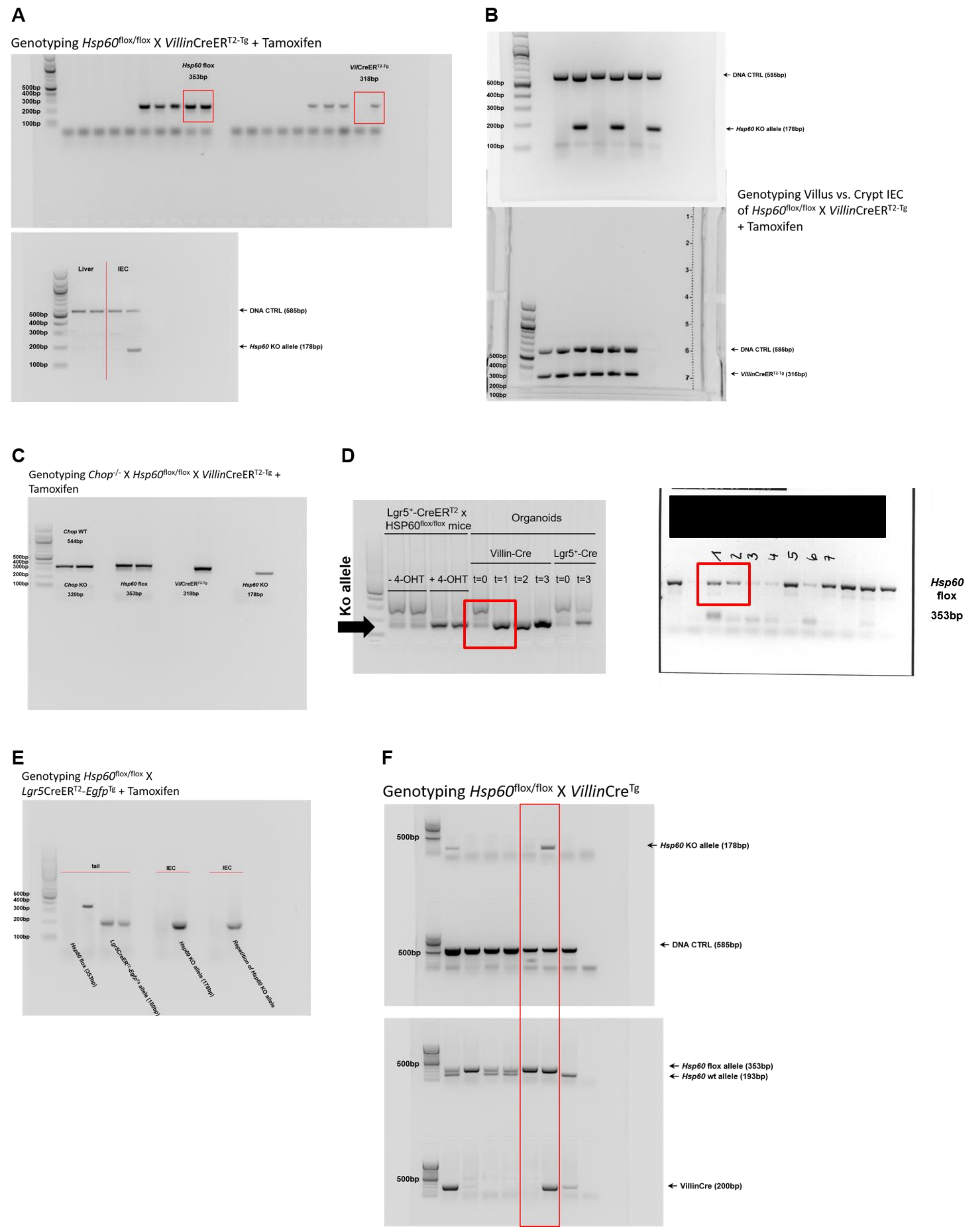

Supplementary Figure 11: Uncropped versions of agarose gels from the main figures

Uncropped versions of agarose gels shown in (A) Figure 1B, (B) Figure 1D, (C) Figure 2, (D) Figure 4C, (E) Figure 6A, (F) Supplementary Figure 1B.

Supplementary Table 1. Primer sequences for genotyping

Allele	Primers (5' → 3')	Allele	Primers (5' → 3')
Hsp60 KO	tctgcctgcttcttctgccttca<br>accagaacaacctcaggcctcaat	*Villin*Cre^{Tg}	caagcctggctcgacggcc<br>cgcgaacatcttcaggttct
Hsp60 flox	accaagaccctgtactcttaacc<br>aacttgacctagatgttgtgtgg	*Villin*CreER$^{T2\text{-}Tg}$	accatatccaccgagtc<br>aggaatgcgatgaagtag
Chop KO	atgcccttacctatcgtg<br>aacgccagggttttcccagtca	*Lgr5*CreER^{T2}-*Egfp*^{Tg}	cactgcattctagttgtgg<br>cggtgcccgcagcgag
Chop WT	atgcccttacctatcgtg<br>gcagggtcaagagtagtg	DNA control	gagactctggctactcatcc<br>ccttcagcaagagctggggac

Supplementary Table 2. Antibodies and dilutions used for IF, IHC and WB

**Primary antibodies:**	**Companies:**	**Dilutions:**
anti-HSP60 (goat /rabbit (WB))	Santa Cruz Biotechnology, Santa Cruz, CA	1:200/ 1:1000 (WB)
anti-Ki67 (rabbit)	abcam, Cambridge, UK	1:400
anti-OLFM4 (rabbit)	Biorbyt, Cambridge, UK	1:200
anti-RSPO1 (rabbit)	Sigma-Aldrich	1:100
anti-GFP (rabbit)	Cell Signalling Technology, Danvers, MA	1:100
anti-E-Cadherin (mouse)	abcam, Cambridge, UK	1:300
anti-αSMA (mouse)	abcam, Cambridge, UK	1:100
anti-WNT10A (rabbit)	abcam, Cambridge, UK	1:100
anti-Lysozyme (goat)	Santa Cruz Biotechnology, Santa Cruz, CA	1:25
anti-Citrate synthase (rabbit)	abcam, Cambridge, UK	1:500 (WB)
anti- β -Actin	Cell Signalling Technology, Danvers, MA	1:1000
anti-8-OHdG (mouse)	abcam, Cambridge, UK	1:1000
Alexa Flour 488 anti-mouse F4/80	Biolegend, San Diego, CA	1:1000

**Secondary antibodies**:	**Companies:**	**Dilutions:**
HRP-conjugated donkey anti-rabbit IgG	dianova, Hamburg, Germany	1:300
HRP-conjugated donkey anti-goat IgG	Sigma-Aldrich, St. Louis, MO	1:300
Biotin-conjugated donkey anti-rabbit IgG	abcam, Cambridge, UK	1:500
Alexa Fluor donkey anti-goat 488/ 546	Life Technologies, Carlsbad, CA	1:200
Alexa Fluor donkey anti-rabbit 546	Life Technologies, Carlsbad, CA	1:200
Alexa Fluor donkey anti-mouse 647	Life Technologies, Carlsbad, CA	1:200

Supplementary Table 3. Primer sequences for quantitative real-time PCR and UPL probe number

Gene	Primers (5' → 3')	Probe	Gene	Primers (5' → 3')	Probe
Hsp60	tcttcaggttgtggcagtca<br>cccctcttctccaaacactg	1	*Wnt7a*	cgctgggagagcgtactg<br>cgataatcgcataggtgaagg	12
Hsp10	ggcccgagttcagagtcc<br>tgtcaaagagcggaagaaactt	77	*Wnt7b*	tctgtccagcggcagttac<br>tcttgttgcagatgatgttgg	49
ClpP	gcaacaagaagcccattcat<br>gtactgcattgtgtcgtagatgg	26	*Wnt8a*	actgcggctgtgacgagt<br>cccgaactccacgttgtc	75
Chop	gcgacagagccagaataaca<br>gatgcacttccttctggaaca	91	*Wnt8b*	gcctcggagactttgacaac<br>ctccccagagccaacctt	76
mtCoxI	cagaccgcaacctaaacaca<br>ttctgggtgcccaaagaat	25	*Wnt9a*	cgagtggacttccacaacaa<br>ggcatttgcaagtggtttc	19
Pgc1α	gagcgaaccttaagtgtggaa<br>tcttggttggctttatgagga	52	*Wnt9b*	ccaagagaggaagcaaggac<br>tctcaggccgctcttcac	105
Otc	gctgtcatggtatccctgct<br>tttccttttgacaggcatca	99	*Wnt10a*	ggcgctcctgttcttccta<br>gtcgttgggtgctgacct	71
Olfm4	gaaattcgagagagagttttctaagg<br>gacctctactcggaccgtca	92	*Wnt10b*	aatgcggatccacaacaac<br>ctccaacaggtcttgaattgg	27
Lgr5	cttcactcggtgcagtgct<br>cagccagctaccaaataggtg	60	*Wnt11*	caggatcccaagccaataaa<br>tccagggaggcacgtaga	94
Axin2	gagagtgagcggcagagc<br>cggctgactcgttctcct	96	*Wnt16*	ccctctttggctatgagctg<br>actgcatgacctggtgacag	94
Wnt1	tactggcactgaccgctct<br>cttggaatccgtcaacaggt	25	*Rspo1*	cgacatgaacaaatgcatca<br>ctcctgacacttggtgcaga	5
Wnt2	cagagatcacagcctctttgg<br>gcgtaaacaaaggccgatt	101	*Tnf*	tgcctatgtctcagcctcttc<br>gaggccatttgggaacttct	49
Wnt2b	ccgggaccacactgtcttt<br>gctgacgagatagcatagacga	16	*Inf*γ	cctttggaccctctgacttg<br>agcgttcattgtctcagagcta	63
Wnt3	ctcgctggctacccaattt<br>gaggccagagatgtgtactgc	81	*Il-6*	gatggatgctaccaaactggat<br>ccaggtagctatggtactccaga	6
Wnt3a	cttagtgctctgcagcctga<br>gagtgctcagagaggagtactgg	76	*Kc*	agactccagccacactccaa<br>tgacagcgcagctcattg	83
Wnt4	actggactccctccctgtct<br>tgcccttgtcactgcaaa	62	*Mcp-1*	catccacgtgttggctca<br>gatcatcttgctggtgaatgagt	62
Wnt5a	acgcttcgcttgaattcct<br>cccgggcttaatattccaa	55	*Ip-10*	gctgccgtcattttctgc<br>tctcactggcccgtcatc	3
Wnt5b	agcaccgtggacaacacat<br>aaggcagtctctcggctacc	53	*Gapdh*	tccactcatggcaaattcaa<br>tttgatgttagtggggtctcg	9
Wnt6	gtgcaactgcacaacaacg<br>ggaacggaggcagcttct	62			
